# scAWMV: an adaptively weighted multi-view learning framework for the integrative analysis of parallel scRNA-seq and scATAC-seq data

**DOI:** 10.1093/bioinformatics/btac739

**Published:** 2022-11-16

**Authors:** Pengcheng Zeng, Yuanyuan Ma, Zhixiang Lin

**Affiliations:** Institute of Mathematical Sciences, ShanghaiTech University, Shanghai 201210, China; School of Computer and Information Engineering, Anyang Normal University, Henan 455000, China; Department of Statistics, The Chinese University of Hong Kong, Hong Kong SAR, China

## Abstract

**Motivation:**

Technological advances have enabled us to profile single-cell multi-omics data from the same cells, providing us with an unprecedented opportunity to understand the cellular phenotype and links to its genotype. The available protocols and multi-omics datasets [including parallel single-cell RNA sequencing (scRNA-seq) and single-cell ATAC sequencing (scATAC-seq) data profiled from the same cell] are growing increasingly. However, such data are highly sparse and tend to have high level of noise, making data analysis challenging. The methods that integrate the multi-omics data can potentially improve the capacity of revealing the cellular heterogeneity.

**Results:**

We propose an adaptively weighted multi-view learning (scAWMV) method for the integrative analysis of parallel scRNA-seq and scATAC-seq data profiled from the same cell. scAWMV considers both the difference in importance across different modalities in multi-omics data and the biological connection of the features in the scRNA-seq and scATAC-seq data. It generates biologically meaningful low-dimensional representations for the transcriptomic and epigenomic profiles via unsupervised learning. Application to four real datasets demonstrates that our framework scAWMV is an efficient method to dissect cellular heterogeneity for single-cell multi-omics data.

**Availability and implementation:**

The software and datasets are available at https://github.com/pengchengzeng/scAWMV.

**Supplementary information:**

Supplementary data are available at *Bioinformatics* online.

## 1 Introduction

The recent advances in single-cell technologies have enabled us to probe multiple biological layers. These technologies include single-cell RNA sequencing (scRNA-seq) that profiles transcription, single-cell ATAC sequencing (scATAC-seq) that profiles accessible chromatin regions (Macaulay *et al.*, 2017; Mezger *et al.*, 2018) and other methods. There are increasing demands for computationally efficient methods for processing and analyzing the datasets (Rotem *et al.*, 2015; Rozenblatt-Rosen *et al.*, 2017) brought by these technologies. For example, clustering methods that group similar cells into sub-populations are often used as the first step in the analysis of single-cell genomic data. The clustering methods for scRNA-seq data include SIMLR (Wang *et al.*, 2017), SC3 (Kiselev *et al.*, 2017), SAFE-clustering (Yang *et al.*, 2018), SOUP (Zhu *et al.*, 2019), SHARP (Wan *et al.*, 2020) and other methods. The clustering methods for scATAC-seq data include Cusanovich 2018 (Cusanovich *et al.*, 2018), scABC (Zamanighomi *et al.*, 2018), SCALE (Xiong *et al.*, 2019), cisTopic (Gonzalez-Blas *et al.*, 2019) and other methods. However, the aforementioned methods are all designed for analyzing one type of genomic feature.

Recently, the technological advances have enabled us to profile high-dimensional multi-omics data at an unprecedented resolution, and the available protocols and multi-omics datasets are growing at an increasing pace. For example, scM&T-seq (Angermueller *et al.*, 2016) links transcriptional and epigenetic heterogeneity, scNMT-seq (Clark *et al.*, 2018) enables joint profiling of chromatin accessibility, DNA methylation and transcription in single cells, and more protocols have been developed for joint profiling of chromatin and transcriptome, including sci-CAR-seq (Cao *et al.*, 2018), scCAT-seq (Liu *et al.*, 2019), Paired-seq (Zhu *et al.*, 2019), SNARE-seq (Chen *et al.*, 2019), SHARE-seq (Ma *et al.*, 2020) and Paired-Tag (Zhu *et al.*, 2021). The resulting single-cell multi-omics datasets can provide insights into the cell’s phenotype and links to its genotype (Macaulay *et al.*, 2017). However, such data tend to have high level of noise and are highly sparse (Colomé-Tatché and Theis, 2018). These characteristics bring challenges for analyzing the multi-omics single-cell data. In order to analyze the complex biological process varying across cells, we need to integrate different types of genomic features via flexible but rigorous computational methods. The methods of data integration are growing recently, and they can be divided into three categories: (i) Non-negative matrix factorization (NMF)-based methods. coupleNMF (Duren *et al.*, 2018) is based on extensions of NMF, and the connection between chromatin accessibility and gene expression builds upon prediction models trained from bulk data with diverse cell types. LIGER (Welch *et al.*, 2019) implements integrative NMF to infer a shared low-dimensional space in multiple single-cell datasets. scAI (Jin *et al.*, 2020) aggregates epigenomic data in cell subpopulations that exhibit similar gene expression and epigenomic profiles through iterative learning in an unsupervised manner. JSNMF (Ma *et al.*, 2022) is based on jointly semi-orthogonal NMF and it enables effective and accurate integrative analysis of single-cell multiomics data. (ii) Methods based on probabilistic generative models. scACE (Lin *et al.*, 2019) is a model-based approach to jointly cluster single-cell chromatin accessibility and single-cell gene expression data. It does not rely on training data to connect the two data types and allows for statistical inference of the cluster assignment. MOFA+ (Argelaguet *et al.*, 2020) is based on extensions of factor analysis model and was designed to deal with increasingly large-scale multi-omics datasets. scAMACE (Wangwu *et al.*, 2021) is a model-based approach to the joint analysis of single-cell data on chromatin accessibility, gene expression and methylation, and it develops an efficient expectation–maximization algorithm to perform statistical inference. (iii) Other machine learning-based methods. Seurat (version 3) (Stuart *et al.*, 2019) anchors diverse datasets together with the capability of integrating single-cell measurements not only across scRNA-seq technologies, but also across different data modalities, that is, data types within single cells, for example, scRNA-seq data, scATAC-seq data and DNA methylation data. coupleCoC (Zeng *et al.*, 2020) and coupleCoC+ (Zeng and Lin, 2021) are transfer learning methods based on the information-theoretic co-clustering framework for the integrative analysis of single-cell genomics data. Seurat (version 4) (Hao *et al.*, 2021) introduces the ‘weighted nearest neighbor’ analysis to learn the relative utility of each genomic feature in each cell, enabling an integrative analysis of multi-omics data.

Two important issues should be taken into account while integrating single-cell multi-omics data. The first issue is how much weight should be assigned to each data modality. The information carried by different types of genomic features are complementary and it is desirable to integrate them. However, the data from different types of genomic features can have different levels of noise. As an example, consider the setting where scRNA-seq and scATAC-seq are profiled on the same cells. scATAC-seq data tend to have higher level of noise and higher degree of sparsity, and it will be intuitive to give smaller weight to scATAC-seq data compared with scRNA-seq data, while integrating the two data modalities. Another issue is how to link data from multiple omics in a way that is biologically meaningful. In the above setting, a subset of features in scATAC-seq data are linked with scRNA-seq data, because promoter accessibility/gene activity score are directly linked with gene expression. Effectively connecting the linked features is expected to be helpful in the integrative analysis of multi-omics data.

**Fig. 1.**
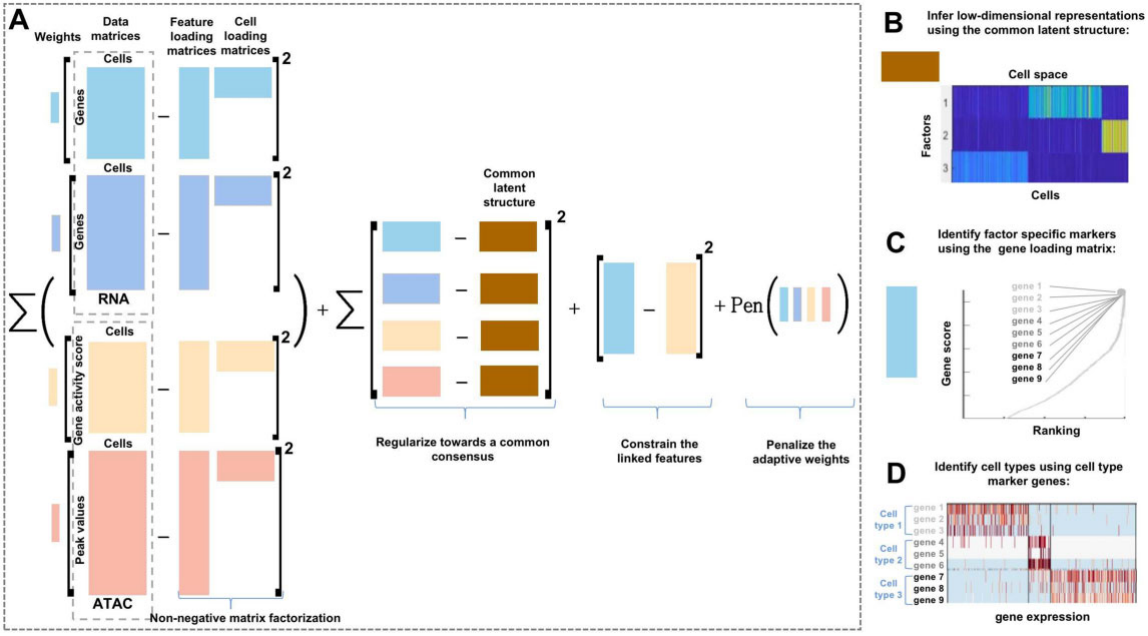
Overview of scAWMV. (**A**) The sketch of the objective function in scAWMV, which is minimized via finding the optimal matrix factorization. It includes four components: (1) reconstruction errors by NMF for the data matrices from scRNA-seq and scATAC-seq, and each factorization is assigned an adaptive weight; (2) the regularization toward a common consensus for all the cell loading matrices; (3) the constraint on the gene loading matrices obtained from the NMF of the linked data, that is, gene expression data matrix in scRNA-seq and gene activity score matrix in scATAC-seq; (4) the penalty term for the adaptive weights. (**B**) Based on the common latent structure from (A), scAWMV uses Louvain clustering and groups the cells in the same clusters in the heatmap of the common latent structure. (**C**) scAWMV ranks genes based on the gene loading matrix for scRNA-seq data from (A). For example, genes 1–9 are labeled with the highest loadings. (**D**) scAWMV assigns cell type labels to cell clusters with known marker genes

In this work, we present scAWMV—an adaptively weighted multi-view learning framework to integrate scRNA-seq data and scATAC-seq data measured from the same cells. Utilizing a unified matrix factorization model, our framework not only automatically assigns a weight to each modality of multi-omics data, but also connects the linked features across data types by adding a constraint (Fig. 1A). Unsupervised learning of this framework will result in biologically meaningful low-rank matrices that represent the transcriptomic and epigenomic profiles. These matrices allow for inferring low-dimensional representations (Fig. 1B), the identification of factor-specific marker genes (Fig. 1C) and the identification of cell types (Fig. 1D). In comparison with the recent multi-omics data integration methods in four published datasets, we demonstrate that our framework is efficient in revealing cellular heterogeneity.

## 2 The methodology

In this section, we first introduce the frameworks of single-view NMF (Wang and Zhang, 2013) and multi-view NMF (Liu *et al.*, 2013), and then extend them to our framework of adaptively weighted multi-view NMF for single-cell multi-omics data. We treat scATAC-seq data and scRNA-seq data that are measured from the same cells as two views of the single-cell genomic data. We assume that a subset of the features, that is, gene activity score in scATAC-seq data is linked with the gene expression in scRNA-seq data; and the other features, that is, accessibility of distal peaks in scATAC-seq data, are not directly linked with the genes in scRNA-seq data. We expect to improve the clustering performance of the cells by (i) integrating these views of data for uncovering the common shared structure; (ii) automatically assigning weights for adjusting for the importance of each view and (iii) adding a constraint to the linked features across data types for considering the biological dependency across different types of genomic features.

### 2.1 Single-view NMF

Let *X* be a *p* by *n* data matrix, representing the data on *p* features of *n* samples. A NMF $X=WH^{T}$ gives us a ‘soft’ clustering of the samples (Duren *et al.*, 2018): the *i*th column of basis matrix *W* gives the mean vectors of the *i*th cluster of samples and the *j*th row of coefficient matrix *H* gives the assignment weights of the *j*th sample to the clusters. The goal of NMF framework (Wang and Zhang, 2013) is to obtain the factorizations by solving the following optimization problem:

(1) $$\underset{W\geq 0,H\geq 0}{\text{argmin}}||X-WH^{T}|{|}_{F}^{2},$$

where $||\cdot |{|}_{F}$ is the Frobenius norm and W≥0,H≥0 stand for the constraints that all elements in the matrix are non-negative.

### 2.2 Multi-view NMF

Assume that the data have *n* views, and let $\left\{X^{\left(1\right)},X^{\left(2\right)},\ldots ,X^{\left(n\right)}\right\}$ denote the data of all the views. Here, for each view $X^{\left(v\right)}$, we have the single-view NMF: $X^{\left(v\right)}=W^{\left(v\right)}{\left(H^{\left(v\right)}\right)}^{T}$. For different views, we have the same number of samples but allow for different number of features. Thus, $H^{\left(v\right)}$s are of the same shape but $W^{\left(v\right)}$s may differ along row dimension across multiple views. To integrate information from multiple views in clustering, Liu *et al.* (2013) developed multi-view NMF framework to cluster multiple views simultaneously to uncover the common latent structure shared by multiple views. A sample in different views would be assigned to the same cluster with high likelihood, thus the coefficient matrices $H^{\left(1\right)},H^{\left(2\right)},\ldots ,H^{\left(n\right)}$ learnt from these views would be required to be softly regularized toward a common consensus $H^{*}$. The goal of multi-view NMF framework (Liu *et al.*, 2013) is to obtain the factorizations in multiple views by solving the following optimization problem:

(2) $$\underset{\overset{W^{\left(v\right)}\geq 0,H^{\left(v\right)}\geq 0,}{H^{*}\geq 0}}{\text{argmin}}\sum_{v=1}^{n}(||X^{\left(v\right)}-W^{\left(v\right)}{\left(H^{\left(v\right)}\right)}^{T}|{|}_{F}^{2}+\lambda^{\left(v\right)}||H^{\left(v\right)}Q^{\left(v\right)}-H^{*}|{|}_{F}^{2}),$$

where $Q^{\left(v\right)}=\text{Diag}(\sum_{i=1}^{p^{\left(v\right)}}W_{i,1}^{\left(v\right)},\sum_{i=1}^{p^{\left(v\right)}}W_{i,2}^{\left(v\right)},\ldots ,\sum_{i=1}^{p^{\left(v\right)}}W_{i,K}^{\left(v\right)})$ is a diagonal matrix; *K* denotes the number of clusters of the samples; $W_{i,k}^{\left(v\right)}$ represents the element of the *i*th row and the *k*th column in the matrix $W^{\left(v\right)}$ and $p^{\left(v\right)}$ denotes the number of features for the *v*th view. Here, $Q^{\left(v\right)}$ is a normalization term for making $H^{\left(v\right)}$ in multiple views comparable at the same scale (Liu *et al.*, 2013). The hyperparameter $\lambda^{\left(v\right)}$ gives the relative weight between the standard NMF reconstruction error $||X^{\left(v\right)}-W^{\left(v\right)}{\left(H^{\left(v\right)}\right)}^{T}|{|}_{F}^{2}$ and the disagreement between the coefficient matrix and the consensus matrix $||H^{\left(v\right)}Q^{\left(v\right)}-H^{*}|{|}_{F}^{2}$ for the *v*th view.

### 2.3 The proposed framework

We now extend the multi-view NMF (Liu *et al.*, 2013) to model single-cell multi-omics data. The gene activity score in scATAC-seq data is linked with gene expression in scRNA-seq data, and the accessibility of distal peaks in scATAC-seq data is not directly linked with the genes in scRNA-seq data. We use the index symbols (v,1) and (v,2) to represent the linked part and the unlinked part of data in the *v*th view, respectively. We write the *v*th view of data as $X^{\left(v\right)}={\left[\begin{array}{c} X^{\left(v,1\right)}\\ X^{\left(v,2\right)} \end{array}\right]}_{p^{\left(v\right)}\times n}$, where $p^{\left(v\right)}=p^{\left(v,1\right)}+p^{\left(v,2\right)}$, representing the sum of the number of features in the linked matrix $X^{\left(v,1\right)}$ and that in the unlinked matrix $X^{\left(v,2\right)}$. Here, we assume that ${\left[X^{\left(1,1\right)}\right]}_{p^{\left(1,1\right)}\times n}$ (i.e. gene expression) is directly linked with ${\left[X^{\left(2,1\right)}\right]}_{p^{\left(2,1\right)}\times n}$ (i.e. gene activity score), and ${\left[X^{\left(1,2\right)}\right]}_{p^{\left(1,2\right)}\times n}$ (i.e. gene expression) is not directly linked with ${\left[X^{\left(2,2\right)}\right]}_{p^{\left(2,2\right)}\times n}$ (i.e. accessibility of distal peaks). We consider these submatrices $X^{\left(1,1\right)},\;X^{\left(1,2\right)},\;X^{\left(2,1\right)}$ and $X^{\left(2,2\right)}$ as the four views of single-cell genomic datasets. We propose the following optimization problem (illustrated in Fig. 1A):

(3) $$\begin{array}{c} \underset{\overset{W^{\left(v,l\right)}\geq 0,H^{\left(v,l\right)}\geq 0,}{H^{*}\geq 0,v,l=1,2}}{\text{argmin}}\sum_{v=1}^{2}\sum_{l=1}^{2}(\omega^{\left(v,l\right)}||X^{\left(v,l\right)}-W^{\left(v,l\right)}{\left(H^{\left(v,l\right)}\right)}^{T}|{|}_{F}^{2}\\ +\lambda^{\left(v,l\right)}||H^{\left(v,l\right)}Q^{\left(v,l\right)}-H^{*}|{|}_{F}^{2})+\beta ||W^{\left(1,1\right)}-W^{\left(2,1\right)}|{|}_{F}^{2}+\gamma \sum_{v=1}^{2}\sum_{l=1}^{2}\omega^{\left(v,l\right)}\text{ln}\omega^{\left(v,l\right)},\\ s.t.\sum_{v=1}^{2}\sum_{l=1}^{2}\omega^{\left(v,l\right)}=1, \end{array}$$

where $Q^{\left(v,l\right)}=\text{Diag}(\sum_{i=1}^{p^{\left(v,l\right)}}W_{i,1}^{\left(v,l\right)},\sum_{i=1}^{p^{\left(v,l\right)}}W_{i,2}^{\left(v,l\right)},\ldots ,\sum_{i=1}^{p^{\left(v,l\right)}}W_{i,K}^{\left(v,l\right)})$, and $W_{i,k}^{\left(v,l\right)}$ represents the element of the *i*th row and the *k*th column in the matrix $W^{\left(v,l\right)}$. Note that $W^{\left(1,1\right)}$ and $W^{\left(2,1\right)}$ have the same number of rows, that is, $p^{\left(1,1\right)}=p^{\left(2,1\right)}$, under the assumption that $X^{\left(1,1\right)}$ is directly linked to $X^{\left(2,1\right)}$, and the number of columns of both $W^{\left(1,1\right)}$ and $W^{\left(2,1\right)}$ is equal to the number of factors *K*.

The naive integration of different views by multi-view NMF does not consider the difference in the relative importance across different views. Therefore, we assign each view $X^{\left(v,l\right)}$ with an adaptive weight $\omega^{\left(v,l\right)}$ in our framework (Equation 3). $\gamma \sum_{v=1}^{2}\sum_{l=1}^{2}\omega^{\left(v,l\right)}\text{ln}\omega^{\left(v,l\right)}$ represents the penalty term for these weights. *γ* is the hyperparameter which controls the distribution of the adaptive weight: when *γ* is smaller, the view that has less reconstruction error NMF (which suggests that the factors carry more effective information) will be given higher weight. More details for the intuition of *γ* and the weights are provided in Section 2.4. The features in the linked data $X^{\left(1,1\right)}$ and $X^{\left(2,1\right)}$ are connected, because gene expression is positively associated with gene activity. We further encourage the difference between the basis matrices $W^{\left(1,1\right)}$ and $W^{\left(2,1\right)}$ to be small. It accounts for the dependence between gene expression in single-cell transcriptomic data and gene activity score in single-cell chromatin accessibility data. The hyperparameter *β* controls the strength of the constraint $||W^{\left(1,1\right)}-W^{\left(2,1\right)}|{|}_{F}^{2}$. The rules for choosing the hyperparameters $\lambda^{\left(v,l\right)}(v,l=1,2)$, *β*, *γ* and the number of factors *K* will be discussed in Section 2.5. We call this adaptively weighted multi-view learning framework scAWMV for short.

### 2.4 Optimization algorithm

We optimize the objective function in Equation (3) by an iterative update procedure:

$$\begin{array}{c} W_{i,k}^{\left(v,1\right)}\leftarrow W_{i,k}^{\left(v,1\right)}\frac{\omega^{\left(v,1\right)}X^{\left(v,1\right)}H^{\left(v,1\right)}+\lambda^{\left(v,1\right)}\sum_{j=1}^{n}H_{j,k}^{\left(v,1\right)}H_{j,k}^{*}+\beta W_{i,k}^{\left(v^{\prime },1\right)}}{\omega^{\left(v,1\right)}{\left(W^{\left(v,1\right)}{\left(H^{\left(v,1\right)}\right)}^{T}H^{\left(v,1\right)}\right)}_{i,k}+\lambda^{\left(v,1\right)}\sum_{m=1}^{p^{\left(v,1\right)}}W_{m,k}^{\left(v,1\right)}\sum_{j=1}^{n}{\left(H_{j,k}^{\left(v,1\right)}\right)}^{2}+\beta W_{i,k}^{\left(v,1\right)}},\;v^{\prime }=3-v,v=1,2;\\ W_{i,k}^{\left(v,2\right)}\leftarrow W_{i,k}^{\left(v,2\right)}\frac{\omega^{\left(v,2\right)}X^{\left(v,2\right)}H^{\left(v,2\right)}+\lambda^{\left(v,2\right)}\sum_{j=1}^{n}H_{j,k}^{\left(v,2\right)}H_{j,k}^{*}}{\omega^{\left(v,2\right)}{\left(W^{\left(v,2\right)}{\left(H^{\left(v,2\right)}\right)}^{T}H^{\left(v,2\right)}\right)}_{i,k}+\lambda^{\left(v,2\right)}\sum_{m=1}^{p^{\left(v,2\right)}}W_{m,k}^{\left(v,2\right)}\sum_{j=1}^{n}{\left(H_{j,k}^{\left(v,2\right)}\right)}^{2}},\;v=1,2;\\ H_{j,k}^{\left(v,l\right)}\leftarrow H_{j,k}^{\left(v,l\right)}\frac{\omega^{\left(v,l\right)}{\left({\left(X^{\left(v,l\right)}\right)}^{T}W^{\left(v,l\right)}\right)}_{j,k}+\lambda^{\left(v,l\right)}H_{j,k}^{*}}{\omega^{\left(v,l\right)}{\left(H^{\left(v,l\right)}{\left(W^{\left(v,l\right)}\right)}^{T}W^{\left(v,l\right)}\right)}_{j,k}+\lambda^{\left(v,l\right)}H_{j,k}^{\left(v,l\right)}},\;v,l=1,2;\\ \omega^{\left(v,l\right)}=\frac{\;\text{exp}\;\{-\frac{||X^{\left(v,l\right)}-W^{\left(v,l\right)}{\left(H^{\left(v,l\right)}\right)}^{T}|{|}_{F}^{2}}{\gamma }\}}{\sum_{v=1}^{2}\sum_{l=1}^{2}\;\text{exp}\;\{-\frac{||X^{\left(v,l\right)}-W^{\left(v,l\right)}{\left(H^{\left(v,l\right)}\right)}^{T}|{|}_{F}^{2}}{\gamma }\}},\;v,l=1,2;\\ H^{*}=\frac{\sum_{v=1}^{2}\sum_{l=1}^{2}\lambda^{\left(v,l\right)}H^{\left(v,l\right)}Q^{\left(v,l\right)}}{\sum_{v=1}^{2}\sum_{l=1}^{2}\lambda^{\left(v,l\right)}}; \end{array}$$

where ${\left(\cdot \right)}_{i,k}$ represents the element of the *i*th row and the *k*th column in the matrix (·). Both $\omega^{\left(v,l\right)}$ and $H^{*}$ have exact solutions in each iteration. The intuition for $\omega^{\left(v,l\right)}$ and *γ* becomes clear by looking at the update for $\omega^{\left(v,l\right)}$: first, the views that have smaller reconstruction error in NMF (i.e. $||X^{\left(v,l\right)}-W^{\left(v,l\right)}{\left(H^{\left(v,l\right)}\right)}^{T}|{|}_{F}^{2}$) will be given higher weight; second, the hyperparameter *γ* acts as a bandwidth parameter. We stop the iterations when the difference of the loss function (Equation 3) is less than $10^{-4}$ between two adjacent iterations. The details for the derivation of these updating rules are given in the Supplementary Materials.

### 2.5 Selection of hyperparameters

We firstly obtain the initial solutions ${\tilde{W}}^{\left(v,l\right)}$ and ${\tilde{H}}^{\left(v,l\right)}(v,l=1,2)$ by solving the optimization problems:

$$\underset{W^{\left(v,l\right)},H^{\left(v,l\right)}}{\text{argmin}}||X^{\left(v,1\right)}-W^{\left(v,1\right)}{\left(H^{\left(v,1\right)}\right)}^{T}|{|}_{F}^{2},\;v,l=1,2,$$

using an approach similar to that in Liu *et al.* (2013), and use them as the initialization for our optimization problem (Equation 3). We initialize the adaptive weights ${\tilde{\omega }}^{\left(v,l\right)}$ as $\frac{1}{4}$ for v,l=1,2. We fix the value of the hyperparameter $\lambda^{\left(v,l\right)}$ as 0.01 for v,l=1,2, as suggested in multi-view NMF by Liu *et al.* (2013). We choose the hyperparameter

(4) $$\beta =\frac{\sum_{v=1}^{2}\sum_{l=1}^{2}{\tilde{\omega }}^{\left(v,l\right)}||X^{\left(v,l\right)}-{\tilde{W}}^{\left(v,l\right)}{\left({\tilde{H}}^{\left(v,l\right)}\right)}^{T}|{|}_{F}^{2}}{||{\tilde{W}}^{\left(1,1\right)}-{\tilde{W}}^{\left(2,1\right)}|{|}_{F}^{2}},$$

and choose the hyperparameter

(5) $$\gamma =\text{max}\{|\frac{||X^{\left(v,l\right)}-{\tilde{W}}^{\left(v,l\right)}{\left({\tilde{H}}^{\left(v,l\right)}\right)}^{T}|{|}_{F}^{2}}{\text{ln}{\tilde{\omega }}^{\left(v,l\right)}}|;v,l=1,2\}.$$

The number of factors *K* can be determined using an approach similar to Brunet *et al.* (2004). In this work, we set *K *=* *20 in all the real datasets. We will discuss in great detail the sensitivity of the choices of the hyperparameters in Section 3.4.

### 2.6 Evaluation of the clustering results

We first compute the cluster of cells by Louvain method (Blondel *et al.*, 2008) once we obtain the consensus matrix $H^{*}$, and then evaluate the clustering performance by three criteria: normalized mutual information (NMI), adjusted Rand index (ARI) (Christopher *et al.*, 2008) and the Residual Average Gini Index (RAGI) score (Chen *et al.*, 2019).

NMI and ARI require the known ground-truth labels of cells, and we use the cell type labels of gene expression data provided in the real datasets as the ground-truth labels. Assume that *G* is the known cell type labels and *P* is the predicted clustering assignments, we then calculate NMI as

$$\frac{I(G;P)}{\sqrt{E(G)\cdot E(P)}},$$

where I(G;P) represents the mutual information over *G* and *P*, and E(·) represents the entropy. Assume that *n* is the total number of single cells, $n_{Q,i}$ is the number of cells assigned to the *i*th cluster in *Q*, $n_{G,j}$ is the number of cells belonging to the *j*th cell type in *G* and $n_{i,j}$ is the number of overlapping cells between the *i*th cluster in *Q* and the *j*th cell type in *G*. As a corrected-for-chance version of the Rand index, ARI is calculated as

$$\frac{\sum_{ij}\left(\begin{array}{c} n_{i,j}\\ 2 \end{array}\right)-[\sum_{i}\left(\begin{array}{c} n_{Q,i}\\ 2 \end{array}\right)\sum_{j}\left(\begin{array}{c} n_{G,j}\\ 2 \end{array}\right)/\left(\begin{array}{c} n\\ 2 \end{array}\right)]}{\frac{1}{2}[\sum_{i}\left(\begin{array}{c} n_{Q,i}\\ 2 \end{array}\right)+\sum_{j}\left(\begin{array}{c} n_{G,j}\\ 2 \end{array}\right)]-[\sum_{i}\left(\begin{array}{c} n_{Q,i}\\ 2 \end{array}\right)\sum_{j}\left(\begin{array}{c} n_{G,j}\\ 2 \end{array}\right)]/\left(\begin{array}{c} n\\ 2 \end{array}\right)}.$$

Higher values of NMI and ARI indicate better clustering performance.

RAGI score calculates the difference between the variability of marker gene expression across cell clusters and the variability of housekeeping gene expression across cell clusters. We note that no cell type labels are required when computing RAGI score on scRNA-seq data. First, we manually find the marker genes using the CellMarker database developed by Zhang *et al.* (2019) and find the housekeeping genes using the HRT Atlas v1.0 database developed by Hounkpe *et al.* (2021). Second, we compute Gini index (Gini, 1997) for each marker gene and each housekeeping gene. The Gini index measures how imbalanced the expression of a gene is across cell clusters. Third, based on the sets of Gini index values for marker genes and housekeeping genes, we calculate the difference between the mean Gini index for marker genes and the mean Gini index for housekeeping genes, that is, the RAGI score. Higher RAGI score represents better separation of the cell clusters.

### 2.7 Data preprocessing and feature selection

In order to get the linked data, we first compute the gene activity score using the gene scoring approach by Chen *et al.* (2019): the distance-weighted sum of reads (peak values in scATAC-seq data) within or near the region gives the accessibility at each transcription start site. Second, we extract the set of genes that have both gene expression and gene activity score, which can be considered as the linked features. Third, we use the scRNA-seq data to choose 2000 most highly variable genes from this set by R toolkit Seurat (Butler *et al.*, 2018; Stuart *et al.*, 2019). We then obtain the linked data ${\left[X^{\left(1,1\right)}\right]}_{2,000\times n}$ and ${\left[X^{\left(2,1\right)}\right]}_{2,000\times n}$. We also use the R toolkit Seurat to choose 2000 most highly variable genes from the set of genes that are not included in the linked data, and we have the unlinked scRNA-seq data ${\left[X^{\left(1,2\right)}\right]}_{2,000\times n}$. We choose 5000 unlinked features in scATAC-seq data, corresponding to the top 5000 largest summation of peak values over all cells, and we have the unlinked scATAC-seq data ${\left[X^{\left(2,2\right)}\right]}_{5,000\times n}$. We take log transformation for scRNA-seq data to alleviate the effect of extreme values in the data matrices. Before implementing our algorithm, we normalized each of these data matrices, including ${\left[X^{\left(1,1\right)}\right]}_{2,000\times n},{\left[X^{\left(2,1\right)}\right]}_{2,000\times n},{\left[X^{\left(1,2\right)}\right]}_{2,000\times n}$ and ${\left[X^{\left(2,2\right)}\right]}_{5,000\times n}$, to make different views of data comparable by dividing the sum of all the entries within that data matrix.

## 3 Results

In this section, we evaluated our methodology in four real datasets of single-cell multiome ATAC and gene expression, including peripheral blood mononuclear cell (PBMC) from healthy donors—granulocytes removed through cell sorting (one dataset has 2711 cells and another one has 11 898 cells), frozen healthy human brain tissue (3233 cells) and fresh frozen lymph node with B-cell lymphoma (14 566 sorted nuclei). All of these datasets are processed by Cell Ranger ARC 2.0.0 and are available at https://www.10xgenomics.com/. We compared our method scAWMV with scAI (Jin *et al.*, 2020), MOFA+ (Argelaguet *et al.*, 2020), Seurat V4 (Hao *et al.*, 2021) and multi-NMF (Liu *et al.*, 2013). To check whether the constraint on the linked data can improve the clustering performance, we compared a simplified version of scAWMV, where the objective function has no constraint on the linked data (i.e. $\beta ||W^{\left(1,1\right)}-W^{\left(2,1\right)}|{|}_{F}^{2}$) compared with that in scAWMV, and we denote it as scAWMV-no-link. To check whether the adaptive weights on different views can improve the clustering performance, we compared another simplified version of scAWMV, where the objective function has no adaptive weights compared with that in scAWMV, and we denote it as scAWMV-no-weights. To check whether the linked part in the datasets is helpful in clustering the cells, we also considered the case where the gene activity score of scATAC-seq data (i.e. $X^{\left(2,1\right)}$) is removed from the input, and we denote it as scAWMV-no-$X^{\left(2,1\right)}$. Except for Seurat V4 (Hao *et al.*, 2021) which can produce clusters of cells by itself, we implemented Louvain clustering on the low-dimensional representation of cells after dimension reduction by the other baseline methods: the cell loading matrix *H* given by scAI (Jin *et al.*, 2020), the latent factor *Z* given by MOFA+ (Argelaguet *et al.*, 2020) and the consensus $V^{*}$ given by multi-NMF (Liu *et al.*, 2013). (Here, the notations *H*, *Z* and $V^{*}$ are consistent with that in their original publications.) We used the NMI, ARI and RAGI to evaluate the clustering results (Table 1). When computing the cluster memberships of cells in four real datasets by all methods except Seurat V4, we set the numbers of clusters the same as that given by the secondary analysis outputs from https://www.10xgenomics.com/.

**Table 1.** The results of clustering the cells in four real datasets for three examples, evaluated by NMI, ARI and RAGI score

	Example 1A			Example 1B			Example 2			Example 3		
Clustering methods	(*n *=* *2711)			(*n *=* *11 898)			(*n *=* *3233)			(*n *=* *14 566)		
	NMI	ARI	RAGI	NMI	ARI	RAGI	NMI	ARI	RAGI	NMI	ARI	RAGI
scAI	0.65	0.47	0.42	0.62	0.37	0.39	0.64	0.44	0.29	0.47	0.18	0.25
MOFA+	0.60	0.43	0.45	0.58	0.39	0.39	0.54	0.33	0.28	0.42	0.22	0.25
Seurat V4	0.60	0.39	**0.48**	0.60	0.43	**0.40**	0.62	0.40	**0.38**	0.48	0.31	**0.28**
multi-NMF	0.68	0.48	0.44	0.61	0.43	0.36	0.64	0.44	0.32	0.42	0.24	0.24
scAWMV-no-link	0.68	0.53	0.47	0.60	0.34	0.36	0.63	0.43	0.31	0.52	0.35	0.26
scAWMV-no-weight	0.69	0.46	0.43	0.59	0.37	0.39	0.64	0.40	0.31	0.43	0.31	0.25
scAWMV-no-$X^{\left(2,1\right)}$	0.67	0.50	0.46	0.59	**0.45**	0.33	0.66	**0.47**	0.31	0.52	0.30	0.22
scAWMV	**0.71**	**0.54**	**0.48**	**0.64**	0.38	**0.40**	**0.69**	0.45	0.31	**0.54**	**0.35**	**0.28**

*Notes*: NMI and ARI were computed by the cell type labels provided by the analysis of gene expression data on the 10× Genomics website. RAGI was computed using marker genes and housekeeping genes. The bold numbers represent the best clustering results.

### 3.1 Example 1: application to PBMCs from healthy donors

In the first example, we studied the paired single-cell multiome ATAC and gene expression of cryopreserved human PBMCs from two healthy female donors (Examples 1A and 1B). The numbers of cells in Examples 1A and 1B are 2711 and 11 898, respectively. We set the number of clusters in Example 1A as 9 and set the number of clusters in Example 1B as 17 when implementing Louvain method for clustering. These numbers of clusters are given by the analysis of gene expression data on the 10× Genomics website. Examples 1A and 1B in Table 1 show the results of clustering the cells. Our method performs the best in NMI, ARI and RAGI among the eight methods in Example 1A, and performs the best in NMI and RAGI among the eight methods in Example 1B. In both Examples 1A and 1B, the value of RAGI given by Seurat V4 ranks the joint first. On the one hand, both scAWMV-no-link and scAWMV-no-weight have slightly worse clustering results compared with scAWMV. This suggests that the removal of the constraint on the linked data or adaptive weights in the objective function of scAWMV has negative impacts on the clustering performance. On the other hand, when we excluded the data matrix $X^{\left(2,1\right)}$ from the input datasets of our method, the clustering performance worsens. It suggests that incorporating the gene activity score in scATAC-seq data and the connection between gene activity score and gene expression in scRNA-seq data are helpful in improving the result of clustering the cells.

**Fig. 2.**
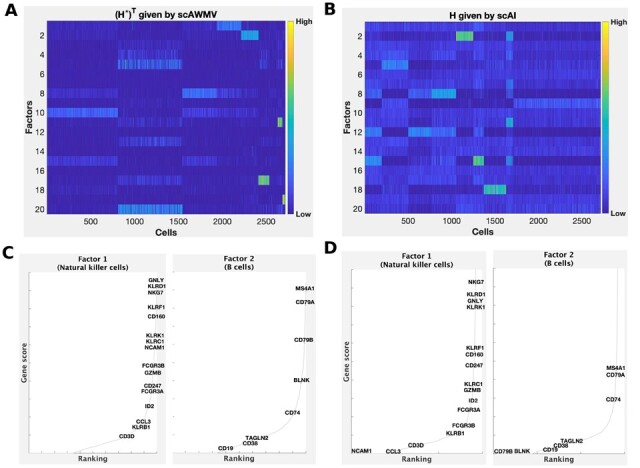
Simultaneously analyzing the paired single-cell multiome ATAC and gene expression for PBMCs in Example 1A. (**A**) Heatmap of the common latent structure ${\left(H^{*}\right)}^{T}$ given by scAWMV. We grouped the cells in the same clusters based on Louvain clustering. (**B**) Heatmap of the latent structure *H* given by scAI. We grouped the cells in the same clusters based on Louvain clustering. (**C**) Ranking of known cell-type-specific marker genes in factors 1 and 2 from $W^{\left(1,1\right)}$ in scAWMV. (**D**) Ranking of known cell-type-specific marker genes in factors 1 and 2 from $W^{\left(2,1\right)}$ in scAWMV. Note that the marker genes for B cells and natural killer cells in PBMCs are collected from CellMarker database (Zhang *et al.*, 2019)

**Fig. 3.**
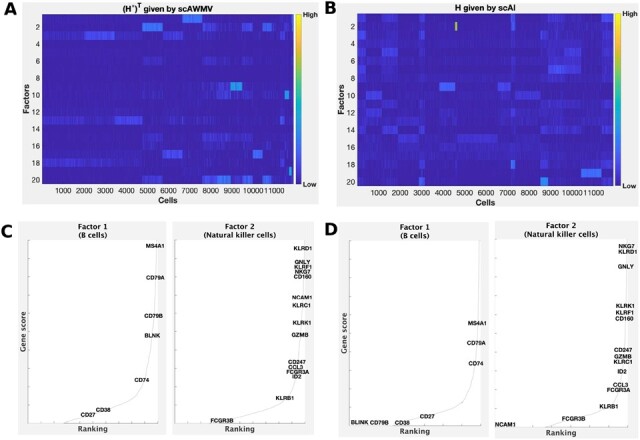
Simultaneously analyzing the paired single-cell multiome ATAC and gene expression for PBMCs in Example 1B. (**A**) Heatmap of the common latent structure ${\left(H^{*}\right)}^{T}$ given by scAWMV. We grouped the cells in the same clusters based on Louvain clustering. (**B**) Heatmap of the latent structure *H* given by scAI. We grouped the cells in the same clusters based on Louvain clustering. (**C**) Ranking of known cell-type-specific marker genes in factors 1 and 2 from $W^{\left(1,1\right)}$ in scAWMV. (**D**) Ranking of known cell-type-specific marker genes in factors 1 and 2 from $W^{\left(2,1\right)}$ in scAWMV. Note that the marker genes for B cells and natural killer cells in PBMCs are collected from CellMarker database (Zhang *et al.*, 2019)

We compared the heatmaps of the common latent structure ${\left(H^{*}\right)}^{T}$ given by scAWMV and the latent structure *H* given by scAI in Figure 2A and B for Example 1A, and in Figure 3A and B for Example 1B, respectively. They show that the consensus ${\left(H^{*}\right)}^{T}$ given by scAWMV can detect more clear patterns than that given by scAI. In Example s1A and 1B, we assigned the cell type labels for all the cell clusters based on marker genes (Supplementary Figs S1 and S2). We then assigned cell types to the factors in the common factor loading matrix ${\left(H^{*}\right)}^{T}$ by the following rule: we selected the top 200 entries in each row of ${\left(H^{*}\right)}^{T}$, calculated the proportions of the cell-type labels corresponding to these selected entries and chose the cell type with the largest proportion as the cell type for the factors. Factors 1 and 2 in Example 1A correspond to natural killer cells (proportion =96%) and B cells (proportion =97%), respectively. Factors 1 and 2 in Example 1B correspond to B cells (proportion =91%) and natural killer cells (proportion =89%), respectively. The marker genes for natural killer cells and B cells (Zhang *et al.*, 2019) also tend to have high scores in the first two factors in $W^{\left(1,1\right)}$ and $W^{\left(2,1\right)}$ (Fig. 2C and D for Example 1A and Fig. 3C and D for Example 1B), which is in agreement with the cell-type annotation for ${\left(H^{*}\right)}^{T}$, and demonstrates that the entries in the feature loading matrices provide biological insight on the factors.

We also studied the gene ontology (GO) enrichment analysis for the feature loading matrices $W^{\left(1,1\right)}$ and $W^{\left(2,1\right)}$, which correspond to gene expression data and gene activity score in chromatin accessibility data, respectively. We selected the top 200 linked genes with large values in each column of $W^{\left(1,1\right)}$ and $W^{\left(2,1\right)}$, and utilized Metascape (Zhou *et al.*, 2019) to implement GO enrichment analysis. The enriched biological processes and pathways for gene expression data and gene activity score tend to be consistent and they agree with the biological function of the underlying cell types that the factors represent (Supplementary Tables S1 and S2). Factor 1 in Example 1A corresponds to natural killer cells, which is a type of cytotoxic lymphocyte critical to the innate immune system that belong to the rapidly expanding family of innate lymphoid cells and represent 5–20% of all circulating lymphocytes in humans (Arachchige and Shavinda, 2021). As demonstrated in Supplementary Table S1, the first columns in $W^{\left(1,1\right)}$ and $W^{\left(2,1\right)}$ are both enriched for ‘cell activation’ (log(q-value) = –14.57 for $W^{\left(1,1\right)}$ and log(q-value) = –10.75 for $W^{\left(2,1\right)}$), ‘regulation of cell activation’ (log(q-value) = –10.84 for $W^{\left(1,1\right)}$ and log(q-value) = –11.31 for $W^{\left(2,1\right)}$) and ‘inflammatory response’ (log(q-value) = –6.24 for $W^{\left(1,1\right)}$ and log(q-value) = –8.29 for $W^{\left(2,1\right)}$). Factor 2 in Example 1A corresponds to B cells, which functions in the humoral immunity component of the adaptive immune system (Murphy, 2012). The second columns in $W^{\left(1,1\right)}$ and $W^{\left(2,1\right)}$ are both enriched for ‘regulation of immune effector process’ (log(q-value) = –5.64 for $W^{\left(1,1\right)}$ and log(q-value) = –9.07 for $W^{\left(2,1\right)}$) and ‘positive regulation of cytokine production’ (log(q-value) = –4.73 for $W^{\left(1,1\right)}$ and log(q-value) = –5.40 for $W^{\left(2,1\right)}$). We observed the same trend in the enrichment analysis for the feature loading matrices $W^{\left(1,1\right)}$ and $W^{\left(2,1\right)}$ in Example 1B (Supplementary Table S2). These results suggest that the feature loading matrices $W^{\left(1,1\right)}$ and $W^{\left(2,1\right)}$ boost GO enrichment analysis and provide rich information on the biological interpretation of the factors. Also, the consistent trends in $W^{\left(1,1\right)}$ and $W^{\left(2,1\right)}$ indicate the effect of the constraint $||W^{\left(1,1\right)}-W^{\left(2,1\right)}|{|}_{F}^{2}$ in scAWMV (Equation 3).

### 3.2 Example 2: application to frozen healthy human brain tissue (3k)

**Fig. 4.**
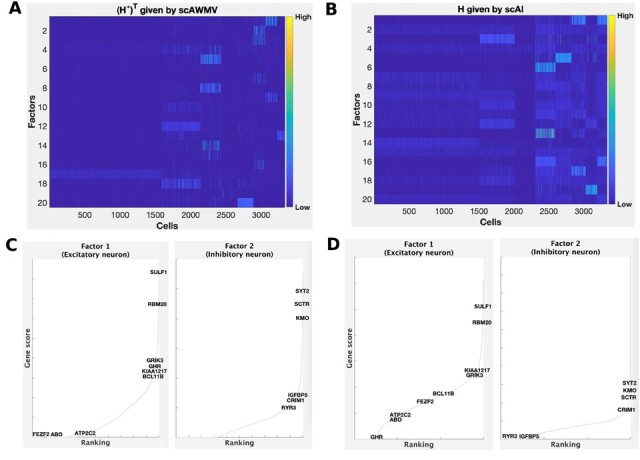
Simultaneously analyzing the paired single-cell multiome ATAC and gene expression for frozen healthy human brain tissue in Example 2. (**A**) Heatmap of the common latent structure ${\left(H^{*}\right)}^{T}$ given by scAWMV. We grouped the cells in the same clusters based on Louvain clustering. (**B**) Heatmap of the latent structure *H* given by scAI. We grouped the cells in the same clusters based on Louvain clustering. (**C**) Ranking of known cell-type-specific marker genes in factors 1 and 2 from $W^{\left(1,1\right)}$ in scAWMV. (**D**) Ranking of known cell-type-specific marker genes in factors 1 and 2 from $W^{\left(2,1\right)}$ in scAWMV. Note that the marker genes for excitatory neuron and inhibitory neuron in human brain are collected from CellMarker database (Zhang *et al.*, 2019)

In the second example, we explored the paired single-cell multiome ATAC and gene expression of flash frozen healthy human brain tissue (cerebellum). The number of cells is 3233. We set the number of clusters as 8, given by the analysis of gene expression data on the 10× Genomics website. The clustering results in Example 2 from Table 1 show that scAWMV performs the best in NMI, scAWMV-no-$X^{\left(2,1\right)}$ performs the best in ARI and Seurat V4 performs the best in RAGI among the eight methods. The clustering performance by scAWMV is slightly better than that by scAWMV-no-link and scAWMV-no-weight. In Figure 4A and B, we compared the heatmaps of the common latent structure ${\left(H^{*}\right)}^{T}$ given by scAWMV and the latent structure *H* given by scAI, respectively. The patterns identified by ${\left(H^{*}\right)}^{T}$ in scAWMV are comparable and slightly clearer than that identified by *H* in scAI. The feature loading matrices $W^{\left(1,1\right)}$ and $W^{\left(2,1\right)}$ given by scAWMV have consistent trends and tend to be enriched for marker genes of the corresponding cell types (Fig. 4C and D). In Figure 4C and D, the enrichment for the modality of chromatin accessibility, $W^{\left(2,1\right)}$, tends to be weaker compared with the modality of gene expression, $W^{\left(1,1\right)}$, which is likely due to the higher level of noise in chromatin accessibility data.

### 3.3 Example 3: application to fresh frozen lymph node with B-cell lymphoma (14k)

**Fig. 5.**
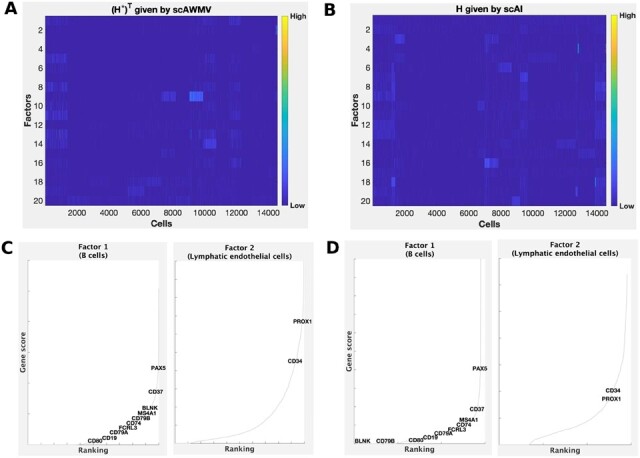
Simultaneously analyzing the paired single-cell multiome ATAC and gene expression for fresh frozen lymph node with B-cell lymphoma in Example 3. (**A**) Heatmap of the common latent structure ${\left(H^{*}\right)}^{T}$ given by scAWMV. We grouped the cells in the same clusters based on Louvain clustering. (**B**) Heatmap of the latent structure *H* given by scAI. We grouped the cells in the same clusters based on Louvain clustering. (**C**) Ranking of known cell-type-specific marker genes in factors 1 and 2 from $W^{\left(1,1\right)}$ in scAWMV. (**D**) Ranking of known cell-type-specific marker genes in factors 1 and 2 from $W^{\left(2,1\right)}$ in scAWMV. Note that the marker genes for B cells and lymphatic endothelial cells in lymph node are collected from CellMarker database (Zhang *et al.*, 2019)

In the last example, we studied the paired single-cell multiome ATAC and gene expression of flash frozen intra-abdominal lymph node tumor from a patient diagnosed with diffuse small lymphocytic lymphoma. The number of cells is 14 566. We set the number of clusters as 18, given by the analysis of gene expression data on the 10× Genomics website. Example 3 in Table 1 shows the results of clustering the cells. Our method scAWMV performs the best in NMI, ARI and RAGI among the eight methods, and the value of RAGI given by Seurat V4 ranks the joint first. Compared with scAWMV, both scAWMV-no-link and scAWMV-no-weight have slightly worse clustering results. The result by scAWMV-no-$X^{\left(2,1\right)}$ shows that the clustering performance of scAWMV becomes worse when we excluded the data matrix $X^{\left(2,1\right)}$ from the input datasets. We compared the heatmaps of the common latent structure ${\left(H^{*}\right)}^{T}$ given by scAWMV and *H* given by scAI in Figure 5A and B. They show that it is hard to detect very clear patterns from the heatmaps of ${\left(H^{*}\right)}^{T}$ and *H*, likely due to the high level of noise in this dataset. In Figure 5C and D, we chose factors 1 and 2 from coefficient matrices $W^{\left(1,1\right)}$ and $W^{\left(2,1\right)}$ given by scAWMV and demonstrate that known cell type markers tend to have higher rankings in the feature loading matrices. In Figure 5C and D, the enrichment for the modality of chromatin accessibility, $W^{\left(2,1\right)}$, tends to be weaker compared with the modality of gene expression, $W^{\left(1,1\right)}$, which is likely due to the higher level of noise in chromatin accessibility data.

### 3.4 Discussion

First, we note that the results from ARI, NMI and RAGI are not be all consistent with each other (Table 1), since the three evaluation criteria depend on different theories and use different inputs: (i) NMI, ARI and RAGI are based on Shannon information, pair-counting and Gini index, respectively, and (ii) both NMI and ARI need the cell type labels as input while RAGI does not. Instead, RAGI needs the list of marker genes and housekeeping genes as input.

Second, in order to study the baseline clustering performance when only one data type, that is, scRNA-seq or scATAC-seq data, is used in all these examples, we compared NMF-only-RNA and NMF-only-ATAC (Supplementary Table S3), which are NMF methods using only scRNA-seq or scATAC-seq data as input. We also summarized the adaptive weights obtained by scAWMV for all views (Supplementary Table S4). The results in Supplementary Table S3 show that the clustering performance of both NMF-only-RNA and NMF-only-ATAC is worse than scAWMV in all the above examples. It suggests that the integration of two data types is needed and adaptive weights need to be adjusted.

Third, we studied the sensitivity of the selection of hyperparameters by scAWMV (Supplementary Figs S3–S7) for the four real datasets in three examples. Supplementary Figure S3 presents the evaluation curves (NMI, ARI and RAGI) as functions of the number of factors *K*, ranging from 10 to 30, for the four real datasets in three examples. It shows that scAWMV performs more stable for Examples 1B and 3, and less stable for Examples 1A and 2. Supplementary Figure S4 presents the evaluation curves with respect to different initializations for the adaptive weights $v=(\omega^{\left(1,1\right)},\omega^{\left(1,2\right)},\omega^{\left(2,1\right)},\omega^{\left(2,2\right)})$, where these initialization values are listed in the figure caption. It shows that the clustering performance by scAWMV is stable for all examples except Example 2, and in all examples, scAWMV has relatively better performance when the adaptive weights are initialized as (1/4,1/4,1/4,1/4). The value λ=0.01 was suggested in multi-view NMF by Liu *et al.* (2013). When we tried different values of *λ*, that is, 0.00001,0.0001,0.001,0.01,0.1,1, scAWMV performs the best when λ=0.01, as shown in Supplementary Figure S5. Supplementary Figures S6 and S7 demonstrate the evaluations with respect to the tuning parameters *β* and *γ*, respectively, where *β*_0_ and *γ*_0_ are the default values calculated from formulas (4) and (5), respectively. They show that scAWMV performs the best when using the default setting $\beta =\beta_{0}$ and $\gamma =\gamma_{0}$. For Examples 1A, 1B and 3, the performance of scAWMV is more stable when *β* and *γ* vary; for Example 2, it is less stable. In summary, except for Example 2, the other three examples are stable to the initialization for the adaptive weights, and the values of *β* and *γ*.

## 4 Convergence and computational time

The algorithm scAWMV is guaranteed to converge as the objective function (Equation 3) is non-increasing in each iteration. It tends to converge in 30 iterations for the four real datasets. We summarized the running time (Supplementary Table S5) by five multi-view clustering methods, including multi-NMF, scAWMV, scAI, MOFA+ and Seurat V4 in each real dataset. The computational costs for Example 1B (∼10K cells) are 36.72 min (multi-NMF), 35.22 min (scAWMV), 600.70 min (scAI), 145.44 min (MOFA+) and 3.96 min (Seurat V4). The graph-based method Seurat V4 is the fastest and our scAWMV is faster or comparable to other methods based on matrix factorization (including multi-NMF, MOFA+ and scAI).

## 5 Conclusion

In this work, we proposed the framework scAWMV for the integrative analysis of parallel scRNA-seq data and scATAC-seq data from the same cells. scAWMV differs from other multi-view learning methods in two aspects: (i) It automatically assigns different weights for different views of data while the other methods tend to treat different views of data equally. (ii) It utilized the linked information between the parallel transcriptomic and epigenomic layers while the other methods tend to ignore this connection. The gene activity score in scATAC-seq data and the gene expression in scRNA-seq data are biologically linked, which may provide useful information in dissecting cellular heterogeneity. Application to four real-world datasets demonstrates that scAWMV is an efficient method to dissect cellular heterogeneity for single-cell multi-omics data.

## Data and software availability

The raw datasets are available at 10× Genomics website https://www.10xgenomics.com/. The software is available at https://github.com/pengchengzeng/scAWMV.

## Supplementary Material

btac739_Supplementary_Data
