# Supplementary material for: scAWMV: an adaptively weighted multi-view learning framework for the integrative analysis of parallel scRNA-seq and scATAC-seq data
Source: Bioinformatics. 2022 Nov 16;39(1):btac739. doi: 10.1093/bioinformatics/btac739 (PMC9805575; doi:10.1093/bioinformatics/btac739)
Supplement: btac739_Supplementary_Data [file btac739_supplementary_data.zip › btac739_Supplementary_Data/scAWMV_supp_final.pdf]

# scAWMV: an Adaptively Weighted Multi-view Learning Framework for the Integrative Analysis of Parallel scRNA-seq Data and scATAC-seq Data

Pengcheng Zeng[1], Yuanyuan Ma[2], and Zhixiang Lin *[3]

[1]Institute of Mathematical Sciences, ShanghaiTech University, Shanghai, China
[2]School of Computuer & Information Engineering, Anyang Normal University, Henan, China
[3]Department of Statistics, The Chinese University of Hong Kong, Hong Kong SAR, China.

November 17, 2022

**SUPPLEMENTARY MATERIAL**

# A. The procedures of algorithm

We denote the objective function in Eq.(3) in the main text as $O$.

(i) Fix $H^{(v,l)}, \omega^{(v,l)}, H^*$, and update $W^{(v,l)}$.

(a) Update $W^{(v,1)}$ by fixing $W^{(v',1)}$ (where $v' = 3 - v$, i.e., $v' = 2$ when $v = 1$ and $v' = 1$ when $v = 2$) and $W^{(v,2)}$. Minimizing the objective function $O$ reduces to minimizing

$$\begin{aligned} f_W^{(v,1)} \triangleq &\omega^{(v,1)}||X^{(v,1)} - W^{(v,1)}(H^{(v,1)})^{\mathrm{T}}||_F^2 + \lambda^{(v,1)}||H^{(v,1)}Q^{(v,1)} - H^*||_F^2 \\ &+ \beta||W^{(v,1)} - W^{(v',1)}||_F^2, \\ &s.t. \quad W^{(v,1)} \geq 0, \end{aligned} \tag{S.1}$$

*Corresponding author: zhixianglin@cuhk.edu.hk

where $Q^{(v,1)} = \mathrm{Diag}\big(\sum_{i=1}^{p^{(v,1)}} W_{i,1}^{(v,1)}, \sum_{i=1}^{p^{(v,1)}} W_{i,2}^{(v,1)}, \ldots, \sum_{i=1}^{p^{(v,1)}} W_{i,K}^{(v,1)}\big)$. We let $\Psi$ be the Lagrange multiplier matrix for the constraint $W^{(v,1)} \geq 0$, and set $L_W^{(v,1)} \triangleq f_W^{(v,1)} + \mathrm{Tr}(\Psi W^{(v,1)})$ be the Lagrange, where $\mathrm{Tr}(\cdot)$ is the trace function. Minimizing $L_W^{(v,1)}$ is equivalent to minimizing

$$\begin{aligned}\hat{L}_W^{(v,1)} \triangleq &\omega^{(v,1)}\mathrm{Tr}\big(W^{(v,1)}(H^{(v,1)})^{\mathrm{T}}H^{(v,1)}(W^{(v,1)})^{\mathrm{T}} - 2X^{(v,1)}H^{(v,1)}(W^{(v,1)})^{\mathrm{T}}\big) + \lambda^{(v,1)}R_W^{(v,1)} \\ &+ \beta\mathrm{Tr}\big(W^{(v,1)}(W^{(v,1)})^{\mathrm{T}} - 2W^{(v,1)}(W^{(v',1)})^{\mathrm{T}}\big) + \mathrm{Tr}(\Psi W^{(v,1)}),\end{aligned} \tag{S.2}$$

where

$$\begin{aligned}R_W^{(v,1)} &\triangleq \mathrm{Tr}\big(H^{(v,1)}Q^{(v,1)}(Q^{(v,1)})^{\mathrm{T}}(H^{(v,1)})^{\mathrm{T}} - 2H^{(v,1)}Q^{(v,1)}(H^{*})^{\mathrm{T}}\big) \\ &= \sum_{j=1}^{n}\sum_{k=1}^{K}\left(H_{j,k}^{(v,1)}\sum_{i=1}^{p^{(v,1)}} W_{i,k}^{(v,1)}\sum_{i=1}^{p^{(v,1)}} W_{i,k}^{(v,1)}H_{j,k}^{(v,1)}\right) \\ &\quad - 2\sum_{j=1}^{n}\sum_{k=1}^{K}\left(H_{j,k}^{(v,1)}\sum_{i=1}^{p^{(v,1)}} W_{i,k}^{(v,1)}H_{j,k}^{*}\right).\end{aligned}$$

Taking derivative of $R_W^{(v,1)}$ with respect to $W^{(v,1)}$ gives

$$S_{i,k} \triangleq \frac{\partial R_W^{(v,1)}}{\partial W_{i,k}^{(v,1)}} = 2\left(\sum_{m=1}^{p^{(v,1)}} W_{m,k}^{(v,1)}\sum_{j=1}^{n}(H_{j,k}^{(v,1)})^2 - \sum_{j=1}^{n} H_{j,k}^{(v,1)}H_{j,k}^{*}\right).$$

Using Karush-Kuhn-Tucker(KKT) conditions, we have

$$\begin{aligned}\frac{\partial \hat{L}_W^{(v,1)}}{\partial W^{(v,1)}} =& -2\omega^{(v,1)}X^{(v,1)}H^{(v,1)} + 2\omega^{(v,1)}W^{(v,1)}(H^{(v,1)})^{\mathrm{T}}H^{(v,1)} + \lambda^{(v,1)}S \\ &+ 2\beta(W^{(v,1)} - W^{(v',1)}) + \Psi = 0; \\ \Psi_{i,k}W_{i,k}^{(v,1)} =& 0, \forall 1 \leq i \leq p^{(v,1)}, 1 \leq k \leq K.\end{aligned}$$

Based on the above conditions, and using the following gradient descent

$$W_{i,k}^{(v,1)} \leftarrow W_{i,k}^{(v,1)} - \tau \cdot \left(\frac{\partial \hat{L}_W^{(v,1)}}{\partial W^{(v,1)}}\right)_{i,k},$$

where the step size $\tau$ is set as

$$\frac{W_{i,k}^{(v,1)}}{2\omega^{(v,1)}\big(W^{(v,1)}(H^{(v,1)})^{\mathrm{T}}H^{(v,1)}\big)_{i,k} + 2\lambda^{(v,1)}\sum_{m=1}^{p^{(v,1)}} W_{m,k}^{(v,1)}\sum_{j=1}^{n}(H_{j,k}^{(v,1)})^2 + 2\beta W_{i,k}^{(v,1)}},$$

and we can derive the following update rule:

$$W_{i,k}^{(v,1)} \leftarrow W_{i,k}^{(v,1)}\frac{\omega^{(v,1)}X^{(v,1)}H^{(v,1)} + \lambda^{(v,1)}\sum_{j=1}^{n} H_{j,k}^{(v,1)}H_{j,k}^{*} + \beta W_{i,k}^{(v',1)}}{\omega^{(v,1)}\big(W^{(v,1)}(H^{(v,1)})^{\mathrm{T}}H^{(v,1)}\big)_{i,k} + \lambda^{(v,1)}\sum_{m=1}^{p^{(v,1)}} W_{m,k}^{(v,1)}\sum_{j=1}^{n}(H_{j,k}^{(v,1)})^2 + \beta W_{i,k}^{(v,1)}}. \tag{S.3}$$

(b) Update $W^{(v,2)}$ by fixing $W^{(v,1)}$. Minimizing the objective function $O$ reduces to minimizing

$$\begin{aligned} f_W^{(v,2)} \triangleq & \omega^{(v,2)}||X^{(v,2)} - W^{(v,2)}(H^{(v,2)})^{\mathrm{T}}||_F^2 + \lambda^{(v,2)}||H^{(v,2)}Q^{(v,2)} - H^*||_F^2, \\ & s.t. \quad W^{(v,2)} \geq 0, \end{aligned} \tag{S.4}$$

where $Q^{(v,2)} = \mathrm{Diag}\big(\sum_{i=1}^{p^{(v,2)}} W_{i,1}^{(v,2)}, \sum_{i=1}^{p^{(v,2)}} W_{i,2}^{(v,2)}, \ldots, \sum_{i=1}^{p^{(v,2)}} W_{i,K}^{(v,2)}\big)$. We let $\Psi$ be the Lagrange multiplier matrix for the constraint $W^{(v,2)} \geq 0$, and set $L_W^{(v,2)} \triangleq f_W^{(v,2)} + \mathrm{Tr}(\Psi W^{(v,2)})$ be the Lagrange. Using the similar way to updating $W^{(v,1)}$, we can derive the following update rule:

$$W_{i,k}^{(v,2)} \leftarrow W_{i,k}^{(v,2)} \frac{\omega^{(v,2)} X^{(v,2)} H^{(v,2)} + \lambda^{(v,2)} \sum_{j=1}^{n} H_{j,k}^{(v,2)} H_{j,k}^*}{\omega^{(v,2)}\big(W^{(v,2)}(H^{(v,2)})^{\mathrm{T}} H^{(v,2)}\big)_{i,k} + \lambda^{(v,2)} \sum_{m=1}^{p^{(v,2)}} W_{m,k}^{(v,2)} \sum_{j=1}^{n} (H_{j,k}^{(v,2)})^2}. \tag{S.5}$$

(ii) Fix $W^{(v,l)}, \omega^{(v,l)}, H^*$, and update $H^{(v,l)}$. Minimizing the objective function $O$ reduces to minimizing

$$\begin{aligned} f_H^{(v,l)} \triangleq & \omega^{(v,l)}||X^{(v,l)} - W^{(v,l)}(H^{(v,l)})^{\mathrm{T}}||_F^2 + \lambda^{(v,l)}||H^{(v,l)}Q^{(v,l)} - H^*||_F^2, \\ & s.t. \quad H^{(v,l)} \geq 0, \end{aligned} \tag{S.6}$$

where $Q^{(v,l)} = \mathrm{Diag}\big(\sum_{i=1}^{p^{(v,l)}} W_{i,1}^{(v,l)}, \sum_{i=1}^{p^{(v,l)}} W_{i,2}^{(v,l)}, \ldots, \sum_{i=1}^{p^{(v,l)}} W_{i,K}^{(v,l)}\big)$. We first normalize $W^{(v,l)}$ and $H^{(v,l)}$ by the following way:

$$W^{(v,l)} \leftarrow W^{(v,l)}(Q^{(v,l)})^{-1}, \quad H^{(v,l)} \leftarrow H^{(v,l)}Q^{(v,l)},$$

and $f_H^{(v,l)}$ is equivalent to

$$\omega^{(v,l)}||X^{(v,l)} - W^{(v,l)}(H^{(v,l)})^{\mathrm{T}}||_F^2 + \lambda^{(v,l)}||H^{(v,l)} - H^*||_F^2. \tag{S.7}$$

Let $\Psi$ be the Lagrange multiplier matrix for the constraint $H^{(v,l)} \geq 0$, and we have the Lagrange as following:

$$\begin{aligned} L_H^{(v,1)} \triangleq & f_H^{(v,l)} + \mathrm{Tr}(\Psi H^{(v,l)}) \\ = & \omega^{(v,l)}\mathrm{Tr}\big(W^{(v,l)}(H^{(v,l)})^{\mathrm{T}} H^{(v,l)}(W^{(v,l)})^{\mathrm{T}} - 2X^{(v,l)}H^{(v,l)}(W^{(v,l)})^{\mathrm{T}}\big) \\ & + \lambda^{(v,l)}\mathrm{Tr}\big(H^{(v,l)}(H^{(v,l)})^{\mathrm{T}} - 2H^{(v,l)}(H^*)^{\mathrm{T}} + \mathrm{Tr}(\Psi H^{(v,l)})\big). \end{aligned} \tag{S.8}$$

Using KKT conditions, we have

$$\begin{aligned} \frac{\partial L_H^{(v,1)}}{\partial H^{(v,l)}} = & -2\omega^{(v,l)}(X^{(v,l)})^{\mathrm{T}} W^{(v,l)} + 2\omega^{(v,l)} H^{(v,l)}(W^{(v,l)})^{\mathrm{T}} W^{(v,l)} \\ & + 2\lambda^{(v,l)}(H^{(v,l)} - H^*) + \Psi = 0; \\ \Psi_{j,k} H_{j,k}^{(v,l)} = & 0, \forall 1 \leq j \leq n, 1 \leq k \leq K. \end{aligned}$$

Based on the above conditions, and using the following gradient descent

$$H_{j,k}^{(v,l)} \leftarrow H_{j,k}^{(v,l)} - \phi \cdot \left( \frac{\partial L_H^{(v,l)}}{\partial H^{(v,l)}} \right)_{j,k},$$

where the step size $\phi$ is set as

$$\frac{H_{j,k}^{(v,l)}}{2\omega^{(v,l)} \big( H^{(v,l)} (W^{(v,l)})^{\mathrm{T}} W^{(v,l)} \big)_{j,k} + 2\lambda^{(v,l)} H_{j,k}^{(v,l)}},$$

and we can derive the following update rule:

$$H_{j,k}^{(v,l)} \leftarrow H_{j,k}^{(v,l)} \frac{\omega^{(v,l)} \big( (X^{(v,l)})^{\mathrm{T}} W^{(v,l)} \big)_{j,k} + \lambda^{(v,l)} H_{j,k}^{*}}{\omega^{(v,l)} \big( H^{(v,l)} (W^{(v,l)})^{\mathrm{T}} W^{(v,l)} \big)_{j,k} + \lambda^{(v,l)} H_{j,k}^{(v,l)}}. \tag{S.9}$$

(iii) Fix $W^{(v,l)}, H^{(v,l)}, H^{*}$, and update $\omega^{(v,l)}$. Minimizing the objective function $O$ reduces to minimizing

$$\begin{aligned} f_\omega \triangleq & \sum_{v=1}^{2} \sum_{l=1}^{2} \omega^{(v,l)} ||X^{(v,l)} - W^{(v,l)} (H^{(v,l)})^{\mathrm{T}}||_F^2 + \gamma \sum_{v=1}^{2} \sum_{l=1}^{2} \omega^{(v,l)} \mathrm{ln} \omega^{(v,l)}, \\ s.t. \quad & \sum_{v=1}^{2} \sum_{l=1}^{2} \omega^{(v,l)} = 1. \end{aligned} \tag{S.10}$$

We then have the Lagrangian function $L_\omega \triangleq f_\omega + \delta \cdot (\sum_{v=1}^{2} \sum_{l=1}^{2} \omega^{(v,l)} - 1)$. By setting $\frac{\partial L_\omega}{\partial \omega^{(v,l)}} = 0$, $\omega^{(v,l)}$ can be analytically expressed as:

$$\omega^{(v,l)} = \frac{\exp\left\{ -\frac{||X^{(v,l)} - W^{(v,l)} (H^{(v,l)})^{\mathrm{T}}||_F^2}{\gamma} \right\}}{\sum_{v=1}^{2} \sum_{l=1}^{2} \exp\left\{ -\frac{||X^{(v,l)} - W^{(v,l)} (H^{(v,l)})^{\mathrm{T}}||_F^2}{\gamma} \right\}}. \tag{S.11}$$

(iv) Fix $W^{(v,l)}, H^{(v,l)}, \omega^{(v,l)}$, and update $H^{*}$. Taking the derivative of the objective function $O$ over $H^{*}$ gives

$$\begin{aligned} \frac{\partial O}{\partial H^{*}} &= \frac{\partial \sum_{v=1}^{2} \sum_{l=1}^{2} \lambda^{(v,l)} ||H^{(v,l)} Q^{(v,l)} - H^{*}||_F^2}{\partial H^{*}} \\ &= \sum_{v=1}^{2} \sum_{l=1}^{2} \lambda^{(v,l)} (-2H^{(v,l)} Q^{(v,l)} + 2H^{*}) = 0. \end{aligned} \tag{S.12}$$

We then have the exact solution for $H^{*}$ by solving Equation (S.12):

$$H^{*} = \frac{\sum_{v=1}^{2} \sum_{l=1}^{2} \lambda^{(v,l)} H^{(v,l)} Q^{(v,l)}}{\sum_{v=1}^{2} \sum_{l=1}^{2} \lambda^{(v,l)}}. \tag{S.13}$$

# B. Figures and tables

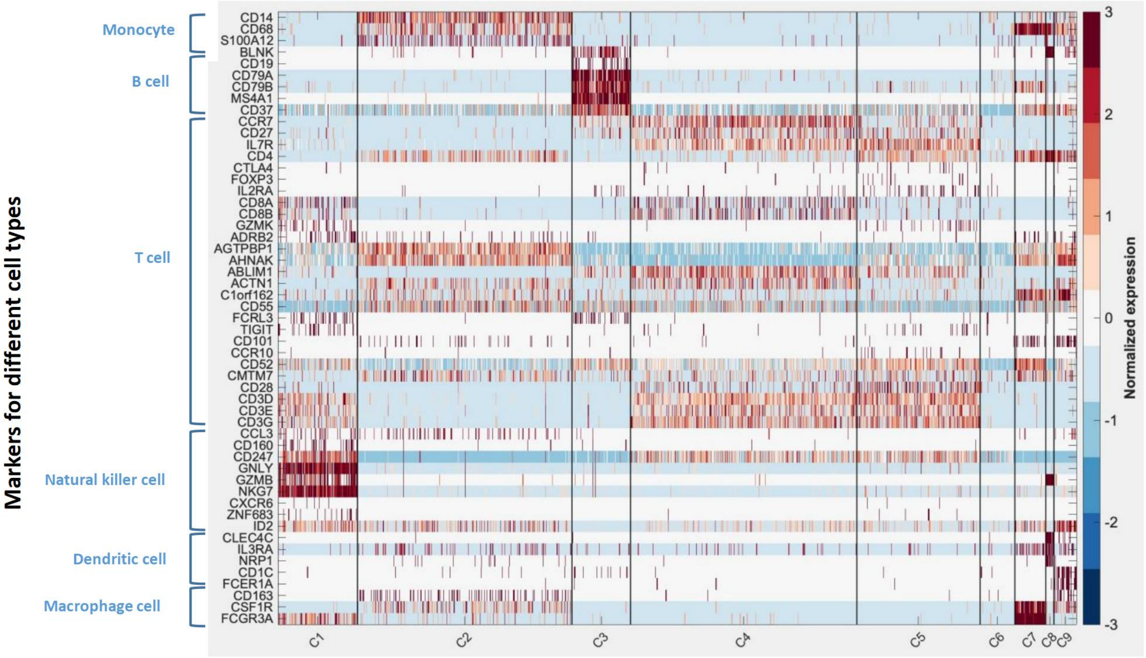


Figure S1: Identifying cell types using cell type marker genes for peripheral blood mononuclear cells in example 1A. It shows the expression of the top 53 marker genes in clusters by scAWMV. We assign the cell type for all the cells in the cluster $C_j$ by firstly computing the value of the average gene expression over markers for each known cell type $k$ in PBMCs (including T cell, B cell, natural killer cell, monocyte cell, macrophage cell and dentritic cell) for all cells in the cluster $C_j$: $Z_{jk} = \frac{\sum_{i \in C_j} (\text{gene expression of the markers for the known cell type k in cell i})}{\#\text{markers for the known cell type k}}, j = 1, \cdots, 9; k = 1, \cdots, 6$, and secondly choosing the cell type $k$ that has the largest value in the set $\{Z_{jk}; k = 1, \cdots, 6.\}$ as the assigned cell type. Based on this rule, the cell types of $C_1, C_2, \cdots, C_9$ are corresponding to natural killer cell, monocyte cell, B cell, T cell, T cell, natural killer cell, macrophage cell, dendritic cell, dendritic cell, respectively.

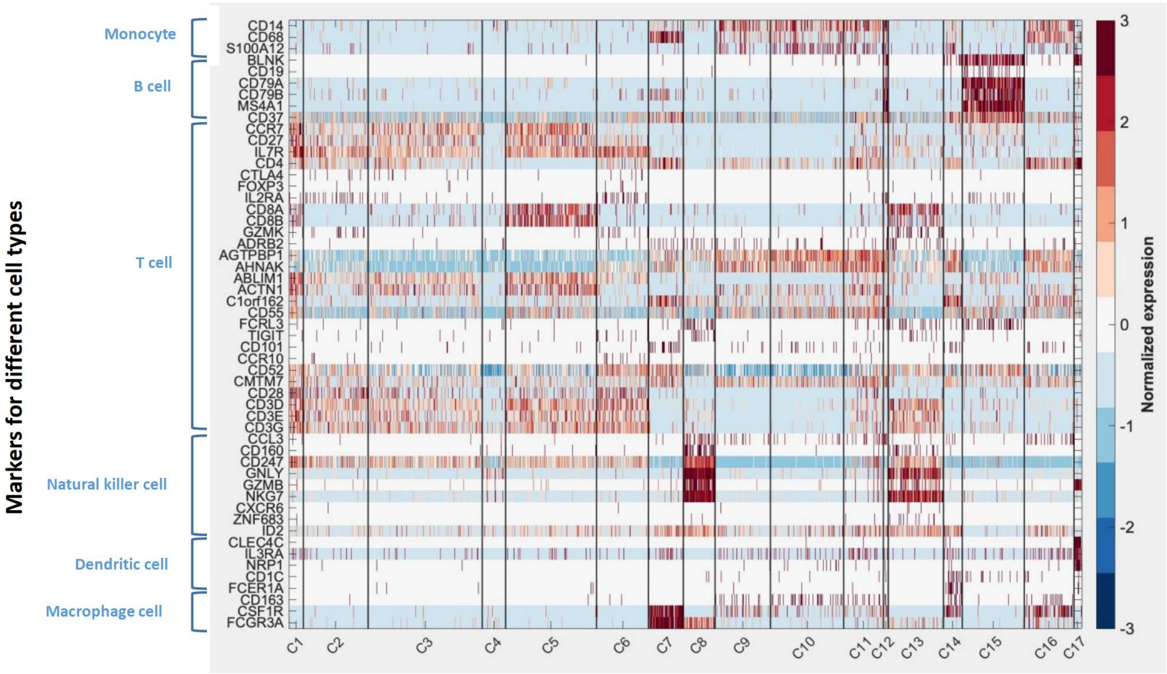


Figure S2: Identifying cell types using cell type marker genes for peripheral blood mononuclear cells in example 1B. It shows the expression of the top 53 marker genes in clusters by scAWMV. We assign the cell type for all the cells in the cluster $C_j$ by firstly computing the value of the average gene expression over markers for each known cell type $k$ in PBMCs (including T cell, B cell, natural killer cell, monocyte cell, macrophage cell and dentritic cell) for all cells in the cluster $C_j$: $Z_{jk} = \frac{\sum_{i \in C_j} (\text{gene expression of the markers for the known cell type k in cell i})}{\text{\#markers for the known cell type k}}, j = 1, \cdots, 17; k = 1, \cdots, 6$, and secondly choosing the cell type $k$ that has the largest value in the set $\{Z_{jk}; k = 1, \cdots, 6.\}$ as the assigned cell type. Based on this rule, cell types of $C_1, C_2, \cdots, C_{17}$ are corresponding to T cell, T cell, T cell, natural killer cell, T cell, T cell, macrophage cell, natural killer cell, T cell, T cell, T cell, B cell, natural killer cell, macrophage cell, B cell, monocyte cell, dendritic cell, respectively.

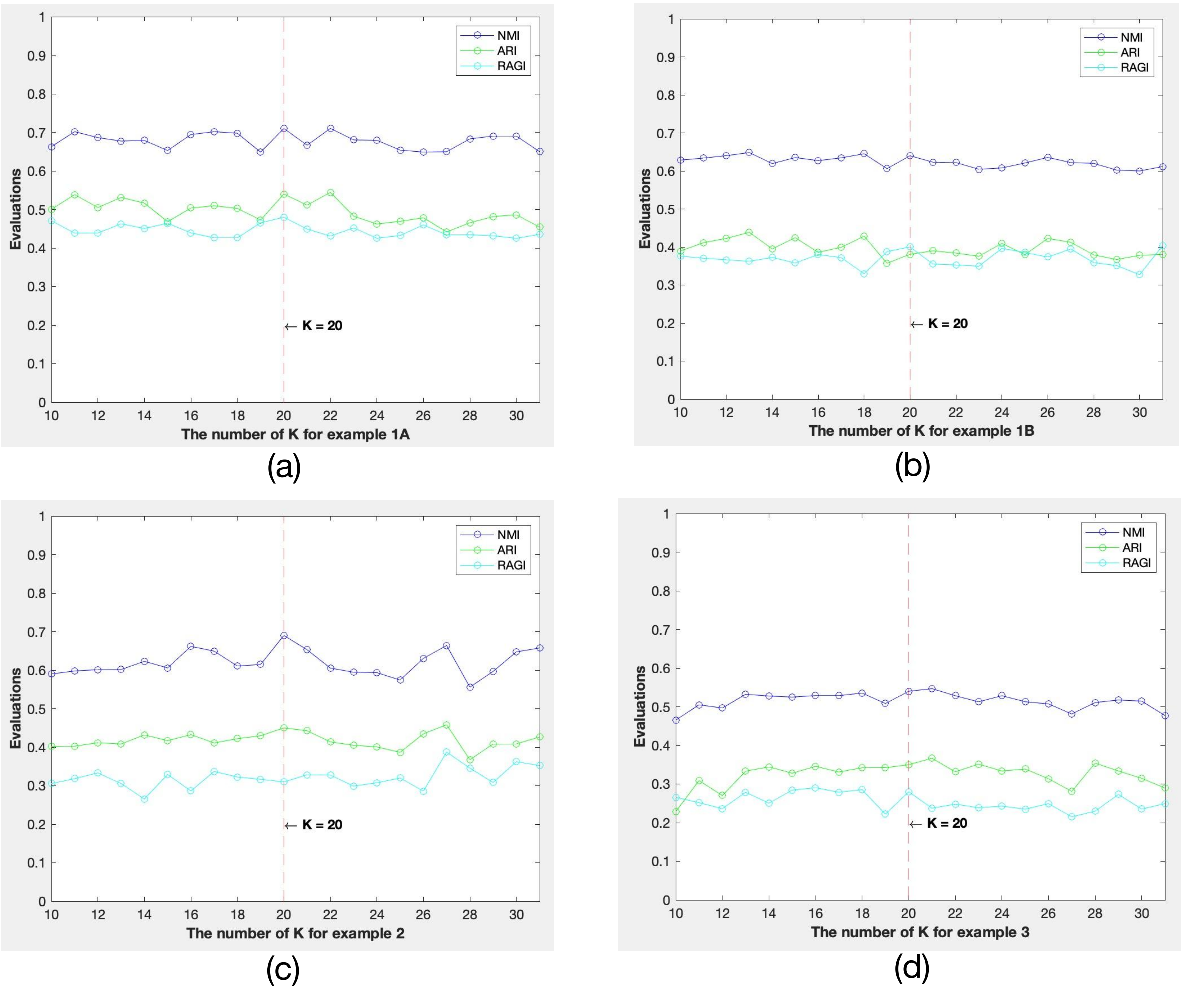


Figure S3: The evaluation curves (NMI, ARI and RAGI) as functions of the number of factors K for the four real datasets in three examples.

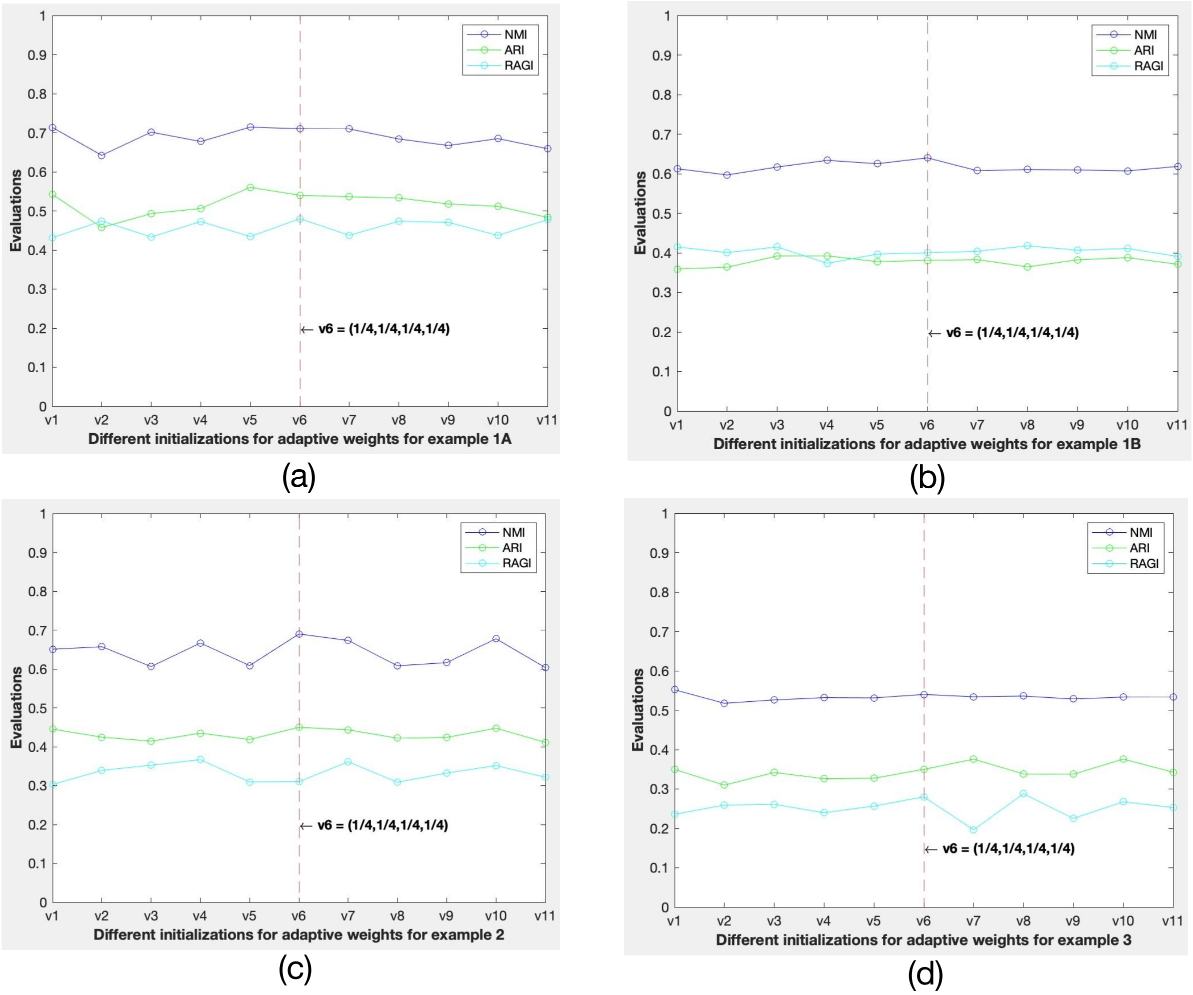


Figure S4: The evaluation curves (NMI, ARI and RAGI) as functions of different initializations for adaptive weights $\boldsymbol{v} = (\omega^{(1,1)}, \omega^{(1,2)}, \omega^{(2,1)}, \omega^{(2,2)})$, where $\boldsymbol{v}1 = (0.25, 0.25, 0.45, 0.05)$, $\boldsymbol{v}2 = (0.25, 0.25, 0.35, 0.15)$, $\boldsymbol{v}3 = (0.25, 0.25, 0.05, 0.45)$, $\boldsymbol{v}4 = (0.25, 0.25, 0.15, 0.35)$, $\boldsymbol{v}5 = (0.45, 0.05, 0.25, 0.25)$, $\boldsymbol{v}6 = (0.25, 0.25, 0.25, 0.25)$, $\boldsymbol{v}7 = (0.35, 0.15, 0.25, 0.25)$, $\boldsymbol{v}8 = (0.05, 0.45, 0.25, 0.25)$, $\boldsymbol{v}9 = (0.15, 0.35, 0.25, 0.25)$, $\boldsymbol{v}10 = (0.45, 0.05, 0.25, 0.25)$, $\boldsymbol{v}11 = (0.05, 0.45, 0.15, 0.35)$, for the four real datasets in three examples.

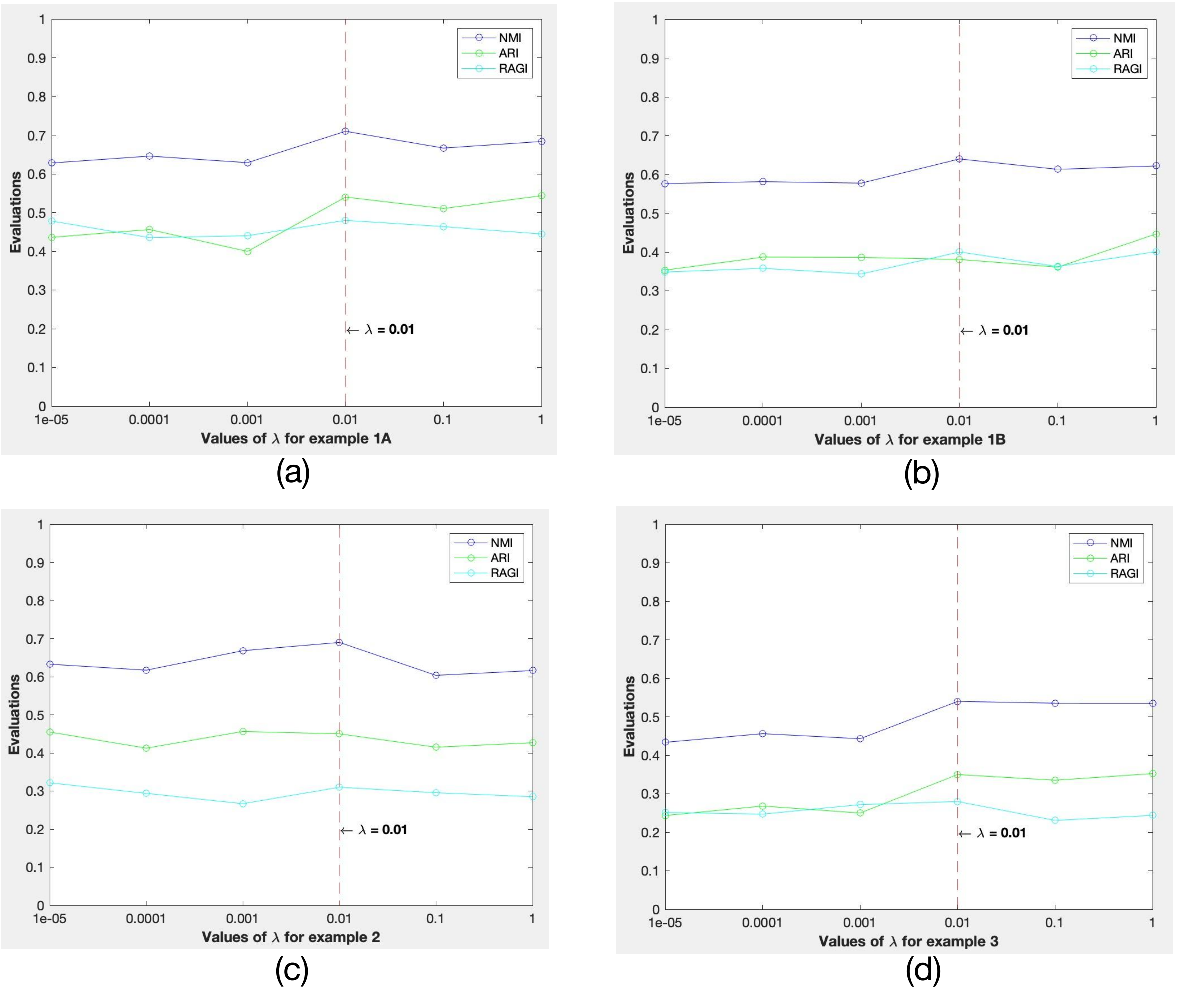


Figure S5: The evaluation curves (NMI, ARI and RAGI) as functions of the value of the tuning parameter $\lambda$ for the four real datasets in three examples.

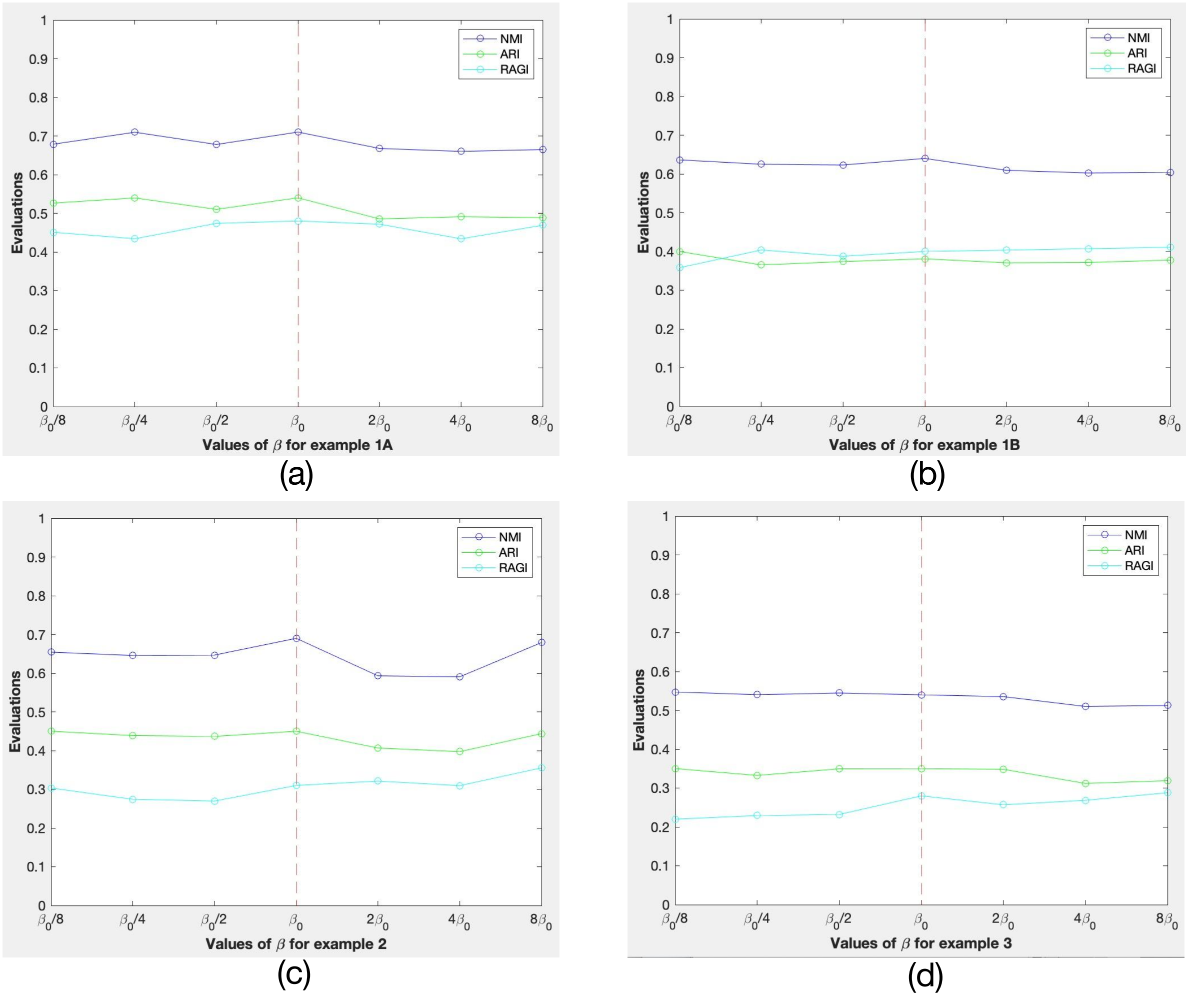


Figure S6: The evaluation curves (NMI, ARI and RAGI) as functions of the value of the tuning parameter $\beta$, where $\beta_0$ is the default value calculated from the formula (4) in the main text, for the four real datasets in three examples.

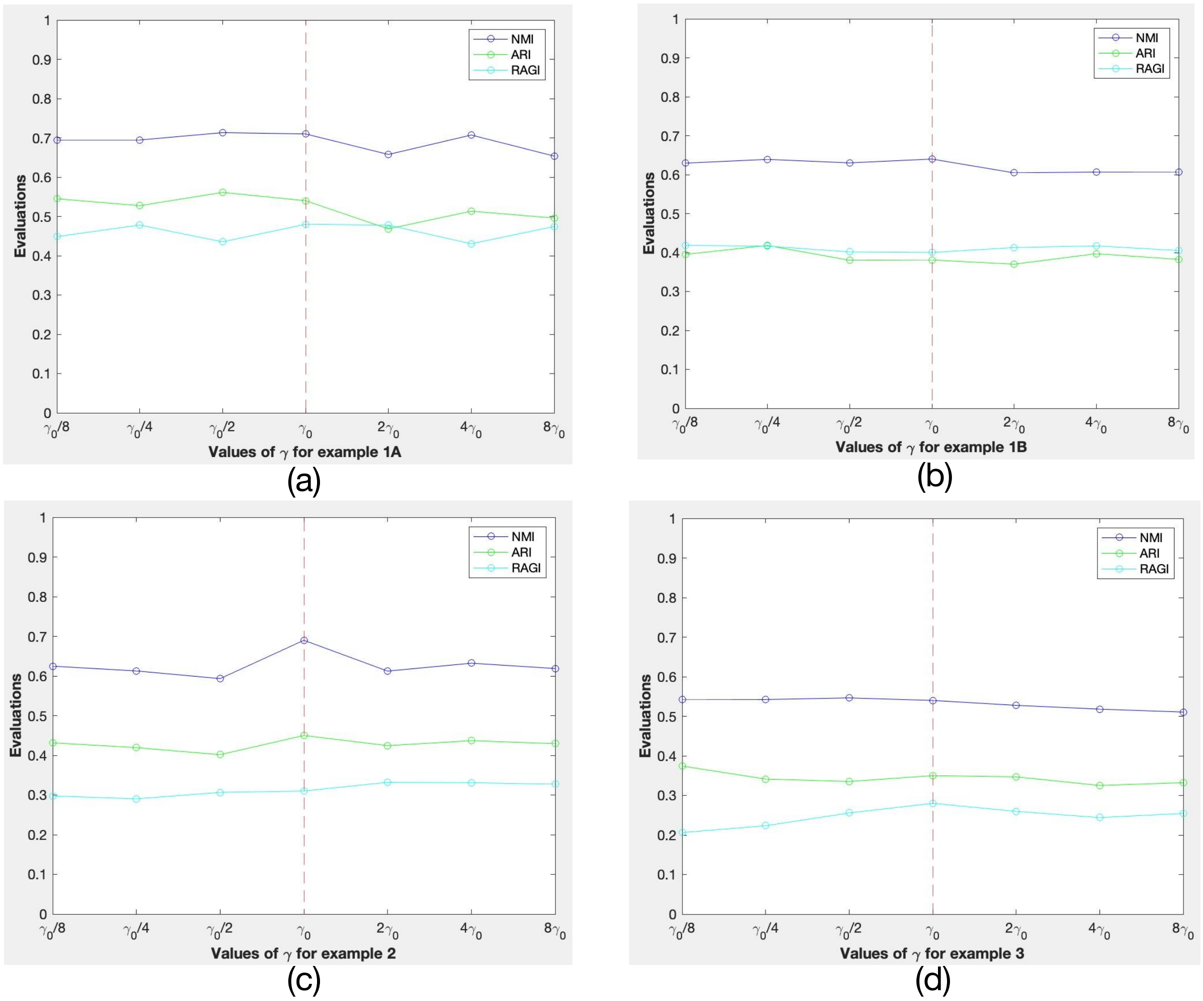


Figure S7: The evaluation curves (NMI, ARI and RAGI) as functions of the value of the tuning parameter $\gamma$, where $\gamma_0$ is the default value calculated from the formula (5) in the main text, for the four real datasets in three examples.

Table S1: Identified significant GO biological processes/pathways in the Metascape for two factors given by scAWMV on peripheral blood mononuclear cells in example 1A. log($q$-value) is the multi-test adjusted $p$-value in log base 10. The pathways are sorted by $q$-value and only the pathways with log($q$-value)$< -4$ are shown.

| Cell type | GO biological process/pathway ($W^{(1,1)}$) | log($q$-value) | GO biological process/pathway ($W^{(2,1)}$) | log($q$-value) |
|---|---|---|---|---|
| Factor 1 Natural killer cells | Cell activation | -14.57 | Regulation of cell activation | -11.31 |
| | Regulation of cell activation | -10.84 | Cell activation | -10.75 |
| | Cell killing | -10.78 | Inflammatory response | -8.29 |
| | Inflammatory response | -6.24 | Immune effector process | -8.08 |
| | cytolysis | -6.05 | Regulation of immune effector process | -7.78 |
| | Positive regulation of programmed cell death | -6 | Regulation of defense response | -7.34 |
| | Cellular response to cytokine stimulus | -5.58 | Cellular response to cytokine stimulus | -5.5 |
| | Response to virus | -5.41 | Response to virus | -4.54 |
| | T cell activation | -5.25 | Nucleosome assembly | -4.4 |
| | Negative regulation of immune system process | -5.18 | Regulation of natural killer cell activation | -4.26 |
| | Cellular defense response | -5.02 | | |
| Factor 2 B cells | Antigen processing and presentation of peptide antigen via MHC class 2 | -19.42 | Leukocyte activation | -9.55 |
| | Regulation of leukocyte proliferation | -12.74 | Regulation of immune effector process | -9.07 |
| | B cell activation | -12.31 | Positive regulation of immune response | -7.37 |
| | Humoral immune response | -5.87 | Cellular response to cytokine stimulus | -6.41 |
| | Regulation of lymphocyte differentiation | -5.82 | Positive regulation of cytokine production | -5.4 |
| | Regulation of immune effector process | -5.64 | Inflammatory response | -4.91 |
| | Response to bacterium | -5.57 | Regulation of MAPK cascade | -4.6 |
| | Ribosome biogenesis | -4.94 | Negative regulation of immune system process | -4.56 |
| | Positive regulation of cytokine production | -4.73 | Regulation of viral entry into host cell | -4.31 |
| | Negative regulation of immune system process | -4.61 | | |

Table S2: Identified significant GO biological processes/pathways in the Metascape for two factors given by scAWMV on peripheral blood mononuclear cells in example 1B. log($q$-value) is the multi-test adjusted $p$-value in log base 10. The pathways are sorted by $q$-value and only the pathways with log($q$-value)$< -4$ are shown.

| Cell type | GO biological process/pathway ($W^{(1,1)}$) | log($q$-value) | GO biological process/pathway ($W^{(2,1)}$) | log($q$-value) |
|---|---|---|---|---|
| Factor 1 B cells | Regulation of leukocyte activation | -18.71 | antigen processing and presentation of exogenous peptide antigen via MHC class II | -14.12 |
| | Immune response-regulating signaling pathway | -17.41 | Regulation of immune effector process | -12.17 |
| | Regulation of leukocyte proliferation | -14.68 | leukocyte activation | -9.97 |
| | Regulation of immune effector process | -9.98 | positive regulation of immune response | -8.67 |
| | Positive regulation of cytokine production | -9.43 | Positive regulation of cytokine production | -8.38 |
| | Cellular response to cytokine stimulus | -6.40 | negative regulation of immune system process | -6.77 |
| | negative regulation of intracellular signal transduction | -6.07 | inflammatory response | -5.52 |
| | regulation of interferon-gamma production | -6.01 | regulation of interferon-gamma production | -4.76 |
| | negative regulation of leukocyte activation | -5.96 | regulation of defense response | -4.72 |
| | positive regulation of cell migration | -5.21 | regulation of response to biotic stimulus | -4.64 |
| | positive regulation of hydrolase activity | -5.03 | negative regulation of intracellular signal transduction | -4.52 |
| Factor 2 Natural killer cells | Cell activation | -18.88 | cell activation | -11.96 |
| | Regulation of cell activation | -15.94 | Regulation of cell activation | -11.19 |
| | positive regulation of immune response | -13.44 | Inflammatory response | -10.36 |
| | Inflammatory response | -9.12 | positive regulation of cytokine production | -10.29 |
| | adaptive immune response | -8.43 | positive regulation of immune response | -9.02 |
| | Cellular response to cytokine stimulus | -8.19 | immune effector process | -7.89 |
| | Fc receptor signaling pathway | -7.74 | regulation of hemopoiesis | -6.07 |
| | positive regulation of cell migration | -7.71 | Fc receptor signaling pathway | -4.88 |
| | cellular defense response | -6.31 | regulation of mononuclear cell migration | -4.57 |
| | response to inorganic substance | -6.24 | Regulation of natural killer cell activation | -4.13 |
| | cytolysis | -6.05 | myeloid leukocyte activation | -4.03 |
| | granulocyte chemotaxis | -5.97 | | |

Table S3: The results of clustering the cells in four real datasets for three examples when only one data type is used, evaluated by NMI, ARI and RAGI score. NMI and ARI were computed by the cell type labels provided by the analysis of gene expression data on the 10X Genomics website. RAGI was computed using marker genes and housekeeping genes.

| Clustering methods | Example 1A ($n = 2,711$) | | | Example 1B ($n = 11,898$) | | | Example 2 ($n = 3,233$) | | | Example 3 ($n = 14,566$) | | |
|---|---|---|---|---|---|---|---|---|---|---|---|---|
| | NMI | ARI | RAGI | NMI | ARI | RAGI | NMI | ARI | RAGI | NMI | ARI | RAGI |
| NMF-only-RNA | 0.67 | 0.48 | 0.44 | 0.62 | 0.34 | 0.35 | 0.61 | 0.42 | 0.29 | 0.53 | 0.31 | 0.23 |
| NMF-only-ATAC | 0.56 | 0.38 | 0.47 | 0.54 | 0.32 | 0.38 | 0.53 | 0.36 | 0.28 | 0.30 | 0.17 | 0.27 |

Table S4: The adaptive weights by scAWMV in three examples.

| Weights | Example 1A ($n = 2,711$) | Example 1B ($n = 11,898$) | Example 2 ($n = 3,233$) | Example 3 ($n = 14,566$) |
|---|---|---|---|---|
| $\omega^{(1,1)}$ | 0.2838 | 0.2781 | 0.2906 | 0.2921 |
| $\omega^{(1,2)}$ | 0.2257 | 0.2325 | 0.1674 | 0.1796 |
| $\omega^{(2,1)}$ | 0.1757 | 0.1759 | 0.1944 | 0.1927 |
| $\omega^{(2,2)}$ | 0.3148 | 0.3136 | 0.3476 | 0.3356 |

Table S5: The computational time by five multi-view clustering methods in three examples. We run our method on an iMac platform with processor - 3.4 GHz Intel Core i5 and memory - 8 GB 2400 MHz DDR4. Multi-NMF, scAWMV and scAI are implemented on MATLAB (version R2020b), and both MOFA+ and Seurat V4 are implemented on RSdudio (version 2021.09.1).

| Clustering methods | Example 1A ($n = 2,711$) | Example 1B ($n = 11,898$) | Example 2 ($n = 3,233$) | Example 3 ($n = 14,566$) |
|---|---|---|---|---|
| multi-NMF | 5.18(min) | 36.72(min) | 5.56(min) | 142.80(min) |
| scAWMV | 5.12(min) | 35.22(min) | 5.41(min) | 124.80(min) |
| scAI | 5.30(min) | 600.70(min) | 6.93(min) | 1540.17(min) |
| MOFA+ | 18.65(min) | 145.44(min) | 56.20(min) | 137.40(min) |
| Seurat V4 | 1.90(min) | 3.96(min) | 2.37(min) | 4.20(min) |
