# Supplementary material for: scAWMV: an adaptively weighted multi-view learning framework for the integrative analysis of parallel scRNA-seq and scATAC-seq data
Source: Bioinformatics. 2022 Nov 16;39(1):btac739. doi: 10.1093/bioinformatics/btac739 (PMC9805575; doi:10.1093/bioinformatics/btac739)
Supplement: btac739_Supplementary_Data [file btac739_supplementary_data.zip › btac739_Supplementary_Data/supp.zip!/supp/com_ex2_part2.pdf]

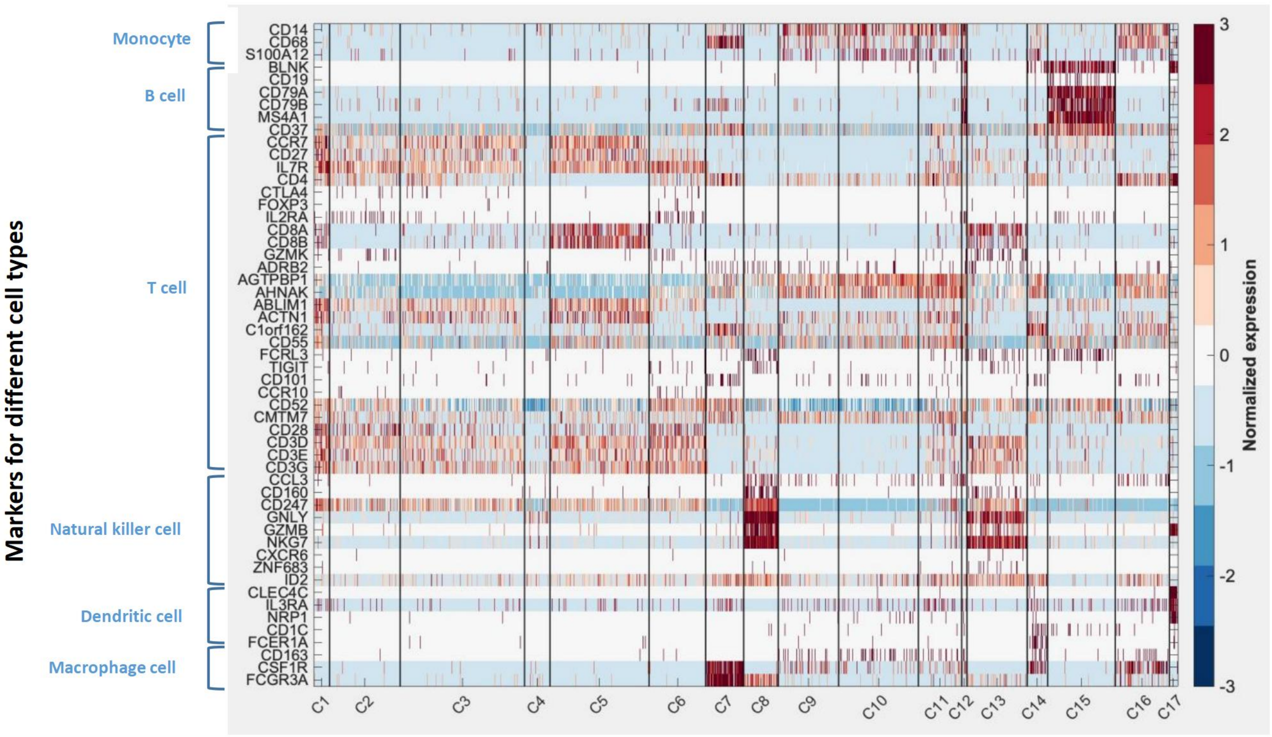
Markers for different cell types
Monocyte
B cell
T cell
Natural killer cell
Dendritic cell
Macrophage cell
CD14
CD68
S100A12
BLNK
CD19
CD79A
CD79B
MS4A1
CD37
CCR7
CD27
IL7R
CD4
CTLA4
FOXP3
IL2RA
CD8A
CD8B
GZMK
ADRB2
AGTPBP1
AHNAK
ABLIM1
ACTN1
C1orf162
CD55
FCRL3
TIGIT
CD101
CCR10
CD52
CMTM7
CD28
CD3D
CD3E
CD3G
CCL3
CD160
CD247
GNLY
GZMB
NKG7
CXCR6
ZNF683
ID2
CLEC4C
IL3RA
NRP1
CD1C
FCER1A
CD163
CSF1R
FCGR3A
C1
C2
C3
C4
C5
C6
C7
C8
C9
C10
C11
C12
C13
C14
C15
C16
C17
3
2
1
0
-1
-2
-3
Normalized expression
