# Supplementary material for: scAWMV: an adaptively weighted multi-view learning framework for the integrative analysis of parallel scRNA-seq and scATAC-seq data
Source: Bioinformatics. 2022 Nov 16;39(1):btac739. doi: 10.1093/bioinformatics/btac739 (PMC9805575; doi:10.1093/bioinformatics/btac739)
Supplement: btac739_Supplementary_Data [file btac739_supplementary_data.zip › btac739_Supplementary_Data/supp.zip!/supp/scAWMV_supp-Copy20220328204257.pdf]

scAWMV: an Adaptively Weighted Multi-view Learning Framework for the Integrative Analysis of Parallel scRNA-seq Data and scATAC-seq Data

Pengcheng Zeng[1] and Zhixiang Lin *[2]

[1]Institute of Mathematical Sciences, ShanghaiTech University, Shanghai, China

[2]Department of Statistics, The Chinese University of Hong Kong, HK

March 28, 2022

SUPPLEMENTARY MATERIAL

A. Bayesian interpretation of $\omega^{(v,l)}$ and γ

The solution to the adaptive weights $\omega^{(v,l)}$ has a Bayesian interpretation from statistical perspective. Let Z be a discrete random variable, taking values in $\{1, 2, 3, 4\}$, where $Z = i$ represents that the observed view is the ith view ($i = 1, 2, 3, 4$). We give Z a prior distribution:

$$P(Z = i) = \frac{1}{4}$$

, which means that each view of data is equally likely to be observed. We define the square root of reconstruction error by NMF as an random variable y, and we have

$$y|_{Z=i} = \sqrt{||X^{(v,l)} - W^{(v,l)}(H^{(v,l)})^{\mathrm{T}}||_F^2}, \quad v, l = 1, 2,$$

where $(v, l) = \big\{(1, 1), (2, 1), (1, 2), (2, 2)\big\}$ corresponds to four views $i = \{1, 2, 3, 4\}$, respectively. We assume that $y^{(z)}$'s are independent and identically distributed random variables, and each of

*Corresponding author: zhixianglin@cuhk.edu.hk

them has a normal distribution with mean 0 and variance $\frac{\gamma}{2}$. Based on the Bayes theorem and the solution to $\omega^{(v,l)}$ using our optimization algorithm (refer to Eq.(S.12)), we have

$$p(\delta^{(z)}=1|y)=\frac{p(y|\delta^{(z)}=1)p(\delta^{(z)}=1))}{\sum_{z=1}^{4}p(y|\delta^{(z)}=1)p(\delta^{(z)}=1))}=\frac{p(y^{(z)})}{\sum_{z=1}^{4}p(y^{(z)})}=\omega^{(v,l)}. \tag{S.1}$$

Since $E(\delta^{(z)}|y)=p(\delta^{(z)}=1|y)$, the adaptive weights $\omega^{(v,l)}$ is actually the posterior mean of the random variable $\delta^{(z)}$. It explains why we use the $\gamma\sum_{v=1}^{2}\sum_{l=1}^{2}\omega^{(v,l)}\ln\omega^{(v,l)}$ as the penalty term for the adaptive weights in the objective function in Eq.(3) in the main text).

Further, when γ is larger, the distribution of $y^{(v)}$ (its variance is $\frac{\gamma}{2}$) becomes flatter, so the probability density of $y^{(z)}$, i.e., $p(y^{(z)})$, tend to be close for different z, which makes the weights $\omega^{(v,l)}$ closer to each other (from the last equality in Eq.(S.1)). On the other hand, when γ is smaller, the distribution of $y^{(v)}$ is more like a "protruding" bell, and the probability density of $y^{(z)}$ tend to be very large when $y^{(v)}$ is close to zero (it means the reconstruction error by NMF is very small). This causes $\omega^{(v,l)}$ much larger than those views corresponding to large reconstruction error. In other words, the better NMF for the zth view, the larger weights assigned to the zth view.

B. The procedures of algorithm

We denote the objective function in Eq.(3) in the main text as O.

(i) Fix $H^{(v,l)},\omega^{(v,l)},H^*$, and update $W^{(v,l)}$.

(a) Update $W^{(v,1)}$ by fixing $W^{(v',1)}$ (where $v'=3-v$, i.e., $v'=2$ when $v=1$ and $v'=1$ when $v=2$) and $W^{(v,2)}$. Minimizing the objective function O reduces to minimizing

$$\begin{aligned} f_W^{(v,1)} \triangleq &\omega^{(v,1)}||X^{(v,1)}-W^{(v,1)}(H^{(v,1)})^{\mathrm{T}}||_F^2+\lambda^{(v,1)}||H^{(v,1)}Q^{(v,1)}-H^*||_F^2\\ &+\beta||W^{(v,1)}-W^{(v',1)}||_F^2,\\ &s.t.\quad W^{(v,1)}\geq 0, \end{aligned} \tag{S.2}$$

where $Q^{(v,1)}=\mathrm{Diag}\big(\sum_{i=1}^{p^{(v,1)}}W_{i,1}^{(v,1)},\sum_{i=1}^{p^{(v,1)}}W_{i,2}^{(v,1)},\ldots,\sum_{i=1}^{p^{(v,1)}}W_{i,K}^{(v,1)}\big)$. We let Ψ be the Lagrange multiplier matrix for the constraint $W^{(v,1)}\geq 0$, and set $L_W^{(v,1)}\triangleq f_W^{(v,1)}+\mathrm{Tr}(\Psi W^{(v,1)})$ be the Lagrange, where $\mathrm{Tr}(\cdot)$ is the trace function. Minimizing $L_W^{(v,1)}$ is equivalent to minimizing

$$\begin{aligned} \hat{L}_W^{(v,1)} \triangleq &\omega^{(v,1)}\mathrm{Tr}\big(W^{(v,1)}(H^{(v,1)})^{\mathrm{T}}H^{(v,1)}(W^{(v,1)})^{\mathrm{T}}-2X^{(v,1)}H^{(v,1)}(W^{(v,1)})^{\mathrm{T}}\big)+\lambda^{(v,1)}R_W^{(v,1)}\\ &+\beta\mathrm{Tr}\big(W^{(v,1)}(W^{(v,1)})^{\mathrm{T}}-2W^{(v,1)}(W^{(v',1)})^{\mathrm{T}}\big)+\mathrm{Tr}(\Psi W^{(v,1)}), \end{aligned} \tag{S.3}$$

where

$$
\begin{aligned}
R_W^{(v,1)} &\triangleq \mathrm{Tr}\big(H^{(v,1)}Q^{(v,1)}(Q^{(v,1)})^{\mathrm{T}}(H^{(v,1)})^{\mathrm{T}} - 2H^{(v,1)}Q^{(v,1)}(H^{*})^{\mathrm{T}}\big) \\
&= \sum_{j=1}^{n}\sum_{k=1}^{K}\left(H_{j,k}^{(v,1)}\sum_{i=1}^{p^{(v,1)}}W_{i,k}^{(v,1)}\sum_{i=1}^{p^{(v,1)}}W_{i,k}^{(v,1)}H_{j,k}^{(v,1)}\right) \\
&\quad - 2\sum_{j=1}^{n}\sum_{k=1}^{K}\left(H_{j,k}^{(v,1)}\sum_{i=1}^{p^{(v,1)}}W_{i,k}^{(v,1)}H_{j,k}^{*}\right).
\end{aligned}
$$

Taking derivative of $R_W^{(v,1)}$ with respect to $W^{(v,1)}$ gives

$$
S_{i,k} \triangleq \frac{\partial R_W^{(v,1)}}{\partial W_{i,k}^{(v,1)}} = 2\left(\sum_{m=1}^{p^{(v,1)}}W_{m,k}^{(v,1)}\sum_{j=1}^{n}(H_{j,k}^{(v,1)})^2 - \sum_{j=1}^{n}H_{j,k}^{(v,1)}H_{j,k}^{*}\right).
$$

Using Karush-Kuhn-Tucker(KKT) conditions, we have

$$
\begin{aligned}
\frac{\partial \hat{L}_W^{(v,1)}}{\partial W^{(v,1)}} =& -2\omega^{(v,1)}X^{(v,1)}H^{(v,1)} + 2\omega^{(v,1)}W^{(v,1)}(H^{(v,1)})^{\mathrm{T}}H^{(v,1)} + \lambda^{(v,1)}S \\
& + 2\beta(W^{(v,1)} - W^{(v',1)}) + \Psi = 0; \\
\Psi_{i,k}W_{i,k}^{(v,1)} =& 0, \forall 1 \leq i \leq p^{(v,1)}, 1 \leq k \leq K.
\end{aligned}
$$

Based on the above conditions, and using the following gradient descent

$$
W_{i,k}^{(v,1)} \leftarrow W_{i,k}^{(v,1)} - \tau \cdot \left(\frac{\partial \hat{L}_W^{(v,1)}}{\partial W^{(v,1)}}\right)_{i,k},
$$

where the step size τ is set as

$$
\frac{W_{i,k}^{(v,1)}}{2\omega^{(v,1)}\big(W^{(v,1)}(H^{(v,1)})^{\mathrm{T}}H^{(v,1)}\big)_{i,k} + 2\lambda^{(v,1)}\sum_{m=1}^{p^{(v,1)}}W_{m,k}^{(v,1)}\sum_{j=1}^{n}(H_{j,k}^{(v,1)})^2 + 2\beta W_{i,k}^{(v,1)}},
$$

and we can derive the following update rule:

$$
W_{i,k}^{(v,1)} \leftarrow W_{i,k}^{(v,1)}\frac{\omega^{(v,1)}X^{(v,1)}H^{(v,1)} + \lambda^{(v,1)}\sum_{j=1}^{n}H_{j,k}^{(v,1)}H_{j,k}^{*} + \beta W_{i,k}^{(v',1)}}{\omega^{(v,1)}\big(W^{(v,1)}(H^{(v,1)})^{\mathrm{T}}H^{(v,1)}\big)_{i,k} + \lambda^{(v,1)}\sum_{m=1}^{p^{(v,1)}}W_{m,k}^{(v,1)}\sum_{j=1}^{n}(H_{j,k}^{(v,1)})^2 + \beta W_{i,k}^{(v,1)}}. \tag{S.4}
$$

(b) Update $W^{(v,2)}$ by fixing $W^{(v,1)}$. Minimizing the objective function O reduces to minimizing

$$
\begin{aligned}
f_W^{(v,2)} \triangleq& \omega^{(v,2)}||X^{(v,2)} - W^{(v,2)}(H^{(v,2)})^{\mathrm{T}}||_F^2 + \lambda^{(v,2)}||H^{(v,2)}Q^{(v,2)} - H^{*}||_F^2, \\
& s.t. \quad W^{(v,2)} \geq 0,
\end{aligned} \tag{S.5}
$$

where $Q^{(v,2)} = \mathrm{Diag}\big(\sum_{i=1}^{p^{(v,2)}} W_{i,1}^{(v,2)}, \sum_{i=1}^{p^{(v,2)}} W_{i,2}^{(v,2)}, \ldots, \sum_{i=1}^{p^{(v,2)}} W_{i,K}^{(v,2)}\big)$. We let Ψ be the Lagrange multiplier matrix for the constraint $W^{(v,2)} \geq 0$, and set $L_W^{(v,2)} \triangleq f_W^{(v,2)} + \mathrm{Tr}(\Psi W^{(v,2)})$ be the Lagrange. Using the similar way to updating $W^{(v,1)}$, we can derive the following update rule:

$$W_{i,k}^{(v,2)} \leftarrow W_{i,k}^{(v,2)} \frac{\omega^{(v,2)} X^{(v,2)} H^{(v,2)} + \lambda^{(v,2)} \sum_{j=1}^{n} H_{j,k}^{(v,2)} H_{j,k}^{*}}{\omega^{(v,2)} \big(W^{(v,2)} (H^{(v,2)})^{\mathrm{T}} H^{(v,2)}\big)_{i,k} + \lambda^{(v,2)} \sum_{m=1}^{p^{(v,2)}} W_{m,k}^{(v,2)} \sum_{j=1}^{n} (H_{j,k}^{(v,2)})^2}. \tag{S.6}$$

(ii) Fix $W^{(v,l)}, \omega^{(v,l)}, H^*$, and update $H^{(v,l)}$. Minimizing the objective function O reduces to minimizing

$$\begin{aligned} f_H^{(v,l)} \triangleq & \omega^{(v,l)} \| X^{(v,l)} - W^{(v,l)} (H^{(v,l)})^{\mathrm{T}} \|_F^2 + \lambda^{(v,l)} \| H^{(v,l)} Q^{(v,l)} - H^* \|_F^2, \\ s.t. \quad & H^{(v,l)} \geq 0, \end{aligned} \tag{S.7}$$

where $Q^{(v,l)} = \mathrm{Diag}\big(\sum_{i=1}^{p^{(v,l)}} W_{i,1}^{(v,l)}, \sum_{i=1}^{p^{(v,l)}} W_{i,2}^{(v,l)}, \ldots, \sum_{i=1}^{p^{(v,l)}} W_{i,K}^{(v,l)}\big)$. We first normalize $W^{(v,l)}$ and $H^{(v,l)}$ by the following way:

$$W^{(v,l)} \leftarrow W^{(v,l)} (Q^{(v,l)})^{-1}, \quad H^{(v,l)} \leftarrow H^{(v,l)} Q^{(v,l)},$$

and $f_H^{(v,l)}$ is equivalent to

$$\omega^{(v,l)} \| X^{(v,l)} - W^{(v,l)} (H^{(v,l)})^{\mathrm{T}} \|_F^2 + \lambda^{(v,l)} \| H^{(v,l)} - H^* \|_F^2. \tag{S.8}$$

Let Ψ be the Lagrange multiplier matrix for the constraint $H^{(v,l)} \geq 0$, and we have the Lagrange as following:

$$\begin{aligned} L_H^{(v,1)} \triangleq & f_H^{(v,l)} + \mathrm{Tr}(\Psi H^{(v,l)}) \\ = & \omega^{(v,l)} \mathrm{Tr}\big(W^{(v,l)} (H^{(v,l)})^{\mathrm{T}} H^{(v,l)} (W^{(v,l)})^{\mathrm{T}} - 2 X^{(v,l)} H^{(v,l)} (W^{(v,l)})^{\mathrm{T}}\big) \\ & + \lambda^{(v,l)} \mathrm{Tr}\big(H^{(v,l)} (H^{(v,l)})^{\mathrm{T}} - 2 H^{(v,l)} (H^*)^{\mathrm{T}} + \mathrm{Tr}(\Psi H^{(v,l)})\big). \end{aligned} \tag{S.9}$$

Using KKT conditions, we have

$$\begin{aligned} \frac{\partial L_H^{(v,1)}}{\partial H^{(v,l)}} = & -2\omega^{(v,l)} (X^{(v,l)})^{\mathrm{T}} W^{(v,l)} + 2\omega^{(v,l)} H^{(v,l)} (W^{(v,l)})^{\mathrm{T}} W^{(v,l)} \\ & + 2\lambda^{(v,l)} (H^{(v,l)} - H^*) + \Psi = 0; \\ \Psi_{j,k} H_{j,k}^{(v,l)} = & 0, \forall 1 \leq j \leq n, 1 \leq k \leq K. \end{aligned}$$

Based on the above conditions, and using the following gradient descent

$$H_{j,k}^{(v,l)} \leftarrow H_{j,k}^{(v,l)} - \phi \cdot \left(\frac{\partial L_H^{(v,l)}}{\partial H^{(v,l)}} \right)_{j,k},$$

where the step size ϕ is set as

$$\frac{H_{j,k}^{(v,l)}}{2\omega^{(v,l)}\big(H^{(v,l)}(W^{(v,l)})^{\mathrm{T}}W^{(v,l)}\big)_{j,k}+2\lambda^{(v,l)}H_{j,k}^{(v,l)}},$$

and we can derive the following update rule:

$$H_{j,k}^{(v,l)} \leftarrow H_{j,k}^{(v,l)}\frac{\omega^{(v,l)}\big((X^{(v,l)})^{\mathrm{T}}W^{(v,l)}\big)_{j,k}+\lambda^{(v,l)}H_{j,k}^{*}}{\omega^{(v,l)}\big(H^{(v,l)}(W^{(v,l)})^{\mathrm{T}}W^{(v,l)}\big)_{j,k}+\lambda^{(v,l)}H_{j,k}^{(v,l)}}. \tag{S.10}$$

(iii) Fix $W^{(v,l)}, H^{(v,l)}, H^{*}$, and update $\omega^{(v,l)}$. Minimizing the objective function O reduces to minimizing

$$\begin{aligned} f_{\omega} &\triangleq \sum_{v=1}^{2}\sum_{l=1}^{2}\omega^{(v,l)}\|X^{(v,l)}-W^{(v,l)}(H^{(v,l)})^{\mathrm{T}}\|_F^2+\gamma\sum_{v=1}^{2}\sum_{l=1}^{2}\omega^{(v,l)}\ln\omega^{(v,l)}, \\ s.t. &\quad \sum_{v=1}^{2}\sum_{l=1}^{2}\omega^{(v,l)}=1. \end{aligned} \tag{S.11}$$

We then have the Lagrangian function $L_{\omega} \triangleq f_{\omega}+\delta\cdot(\sum_{v=1}^{2}\sum_{l=1}^{2}\omega^{(v,l)}-1)$. By setting $\frac{\partial L_{\omega}}{\partial\omega^{(v,l)}}=0$, $\omega^{(v,l)}$ can be analytically expressed as:

$$\omega^{(v,l)}=\frac{\exp\Big\{-\frac{\|X^{(v,l)}-W^{(v,l)}(H^{(v,l)})^{\mathrm{T}}\|_F^2}{\gamma}\Big\}}{\sum_{v=1}^{2}\sum_{l=1}^{2}\exp\Big\{-\frac{\|X^{(v,l)}-W^{(v,l)}(H^{(v,l)})^{\mathrm{T}}\|_F^2}{\gamma}\Big\}}. \tag{S.12}$$

(iv) Fix $W^{(v,l)}, H^{(v,l)}, \omega^{(v,l)}$, and update H^{*}. Taking the derivative of the objective function O over H^{*} gives

$$\begin{aligned} \frac{\partial O}{\partial H^{*}} &= \frac{\partial\sum_{v=1}^{2}\sum_{l=1}^{2}\lambda^{(v,l)}\|H^{(v,l)}Q^{(v,l)}-H^{*}\|_F^2}{\partial H^{*}} \\ &= \sum_{v=1}^{2}\sum_{l=1}^{2}\lambda^{(v,l)}(-2H^{(v,l)}Q^{(v,l)}+2H^{*})=0. \end{aligned} \tag{S.13}$$

We then have the exact solution for H^{*} by solving Equation (S.13):

$$H^{*}=\frac{\sum_{v=1}^{2}\sum_{l=1}^{2}\lambda^{(v,l)}H^{(v,l)}Q^{(v,l)}}{\sum_{v=1}^{2}\sum_{l=1}^{2}\lambda^{(v,l)}}. \tag{S.14}$$

C. Comparison of computation time

Table S.1: The computation time by five multi-view clustering methods in four examples.

Clustering methods	Example 1 ($n = 3$k)	Example 2 ($n = 10$k)	Example 3 ($n = 3$k)	Example 4 ($n = 14$k)
multi-NMF	5.18(min)	36.72(min)	5.56(min)	142.80(min)
scAWMV	5.12(min)	35.22(min)	5.41(min)	124.80(min)
scAI	3.98(min)	374.46(min)	10.20(min)	733.80(min)
MOFA+	18.65(min)	145.44(min)	56.20(min)	137.40(min)
Seurat V4	1.90(min)	3.96(min)	2.37(min)	4.20(min)
