# Supplementary material for: scAWMV: an adaptively weighted multi-view learning framework for the integrative analysis of parallel scRNA-seq and scATAC-seq data
Source: Bioinformatics. 2022 Nov 16;39(1):btac739. doi: 10.1093/bioinformatics/btac739 (PMC9805575; doi:10.1093/bioinformatics/btac739)
Supplement: btac739_Supplementary_Data [file btac739_supplementary_data.zip › btac739_Supplementary_Data/supp.zip!/supp/tp2-v4.pdf]

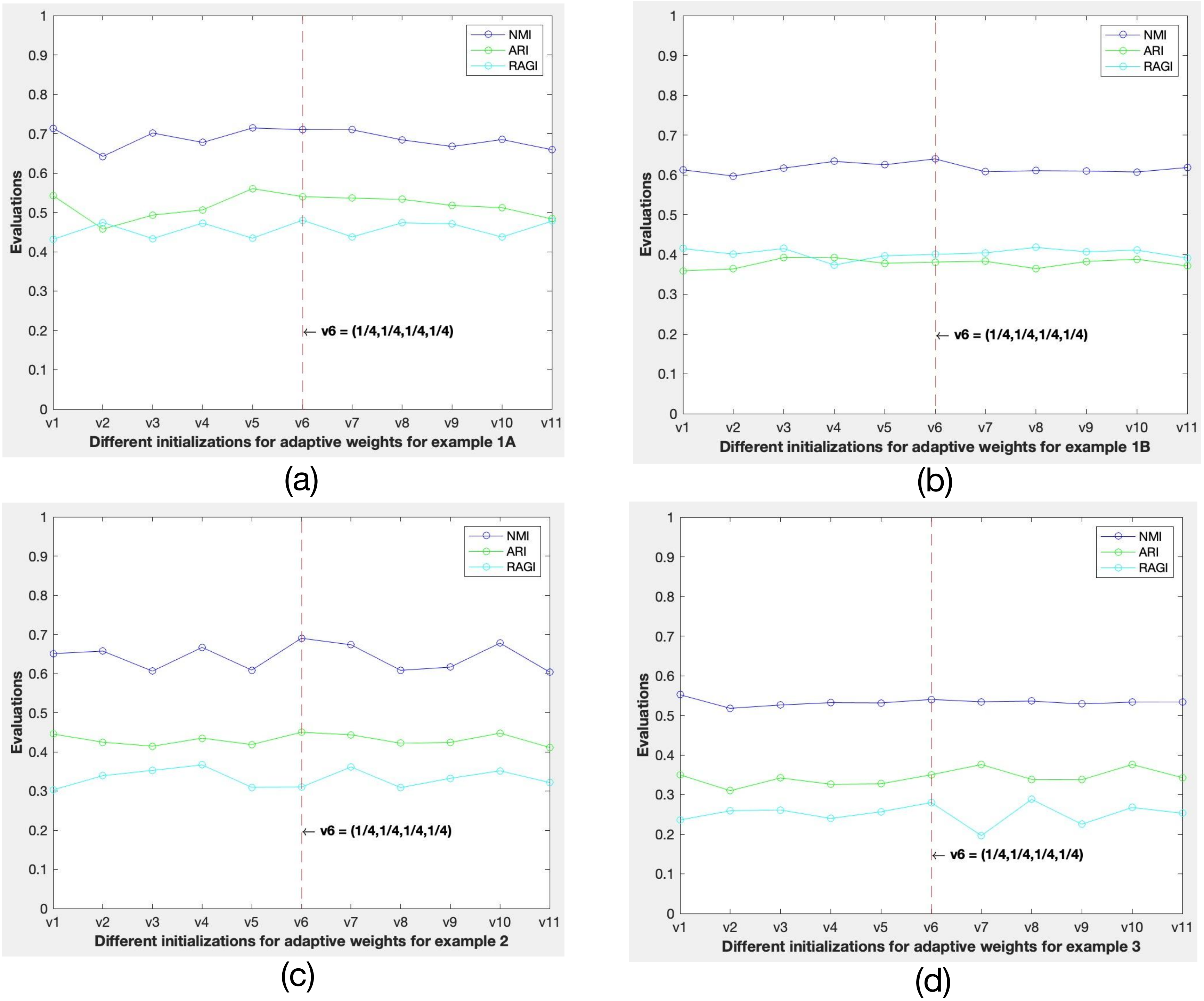

NMI
ARI
RAGI
← v6 = (1/4,1/4,1/4,1/4)
Evaluations
Different initializations for adaptive weights for example 1A
(a)
Different initializations for adaptive weights for example 1B
(b)
Different initializations for adaptive weights for example 2
(c)
Different initializations for adaptive weights for example 3
(d)
