# Supplementary material for: scAWMV: an adaptively weighted multi-view learning framework for the integrative analysis of parallel scRNA-seq and scATAC-seq data
Source: Bioinformatics. 2022 Nov 16;39(1):btac739. doi: 10.1093/bioinformatics/btac739 (PMC9805575; doi:10.1093/bioinformatics/btac739)
Supplement: btac739_Supplementary_Data [file btac739_supplementary_data.zip › btac739_Supplementary_Data/supp.zip!/supp/tp2.pdf]

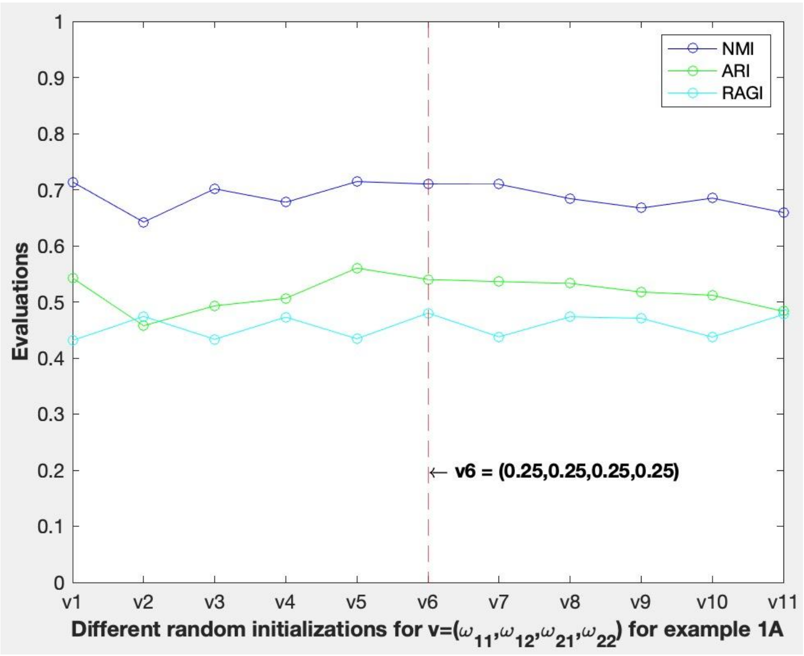
NMI
ARI
RAGI
Evaluations
← v6 = (0.25,0.25,0.25,0.25)
v1 v2 v3 v4 v5 v6 v7 v8 v9 v10 v11
Different random initializations for v=(ω_{11},ω_{12},ω_{21},ω_{22}) for example 1A

(a)

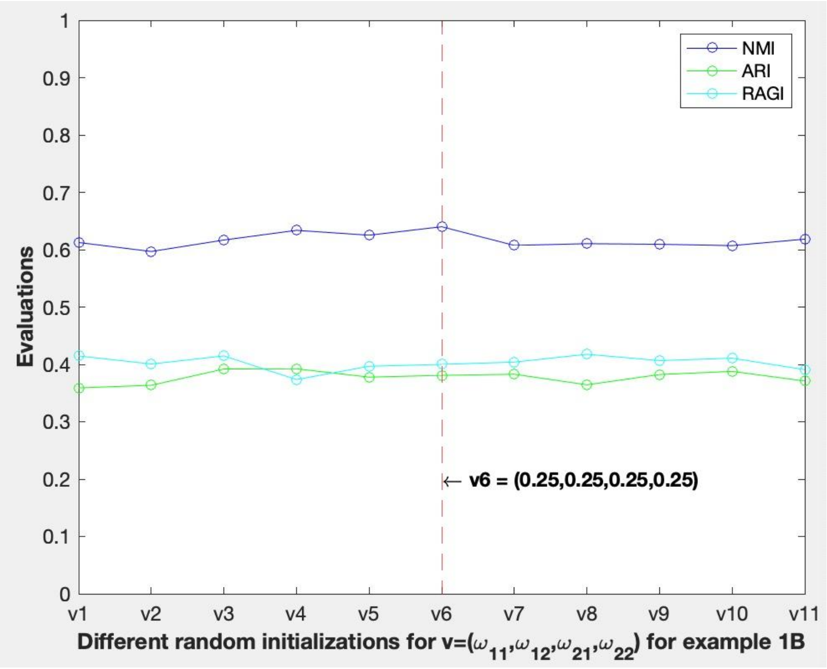
NMI
ARI
RAGI
Evaluations
← v6 = (0.25,0.25,0.25,0.25)
v1 v2 v3 v4 v5 v6 v7 v8 v9 v10 v11
Different random initializations for v=(ω_{11},ω_{12},ω_{21},ω_{22}) for example 1B

(b)

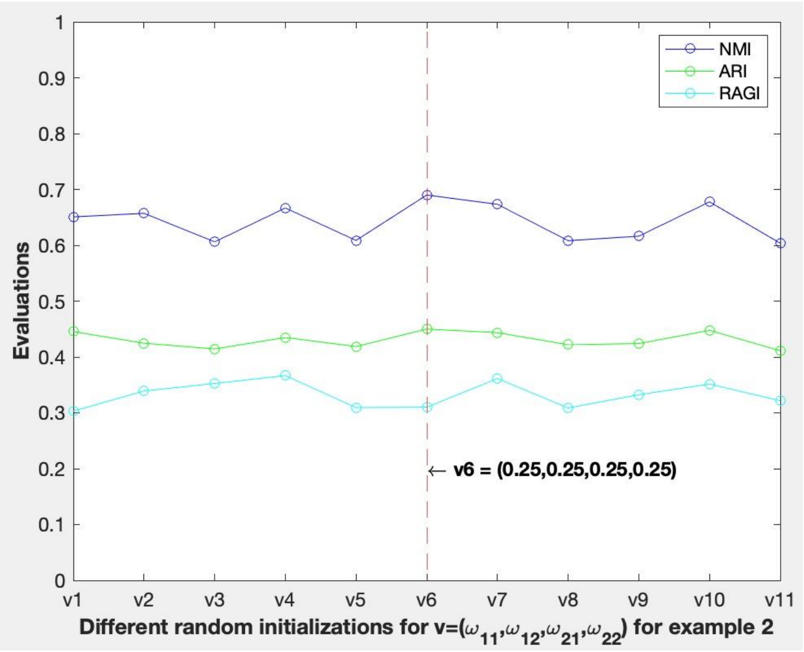
NMI
ARI
RAGI
Evaluations
← v6 = (0.25,0.25,0.25,0.25)
v1 v2 v3 v4 v5 v6 v7 v8 v9 v10 v11
Different random initializations for v=(ω_{11},ω_{12},ω_{21},ω_{22}) for example 2

(c)

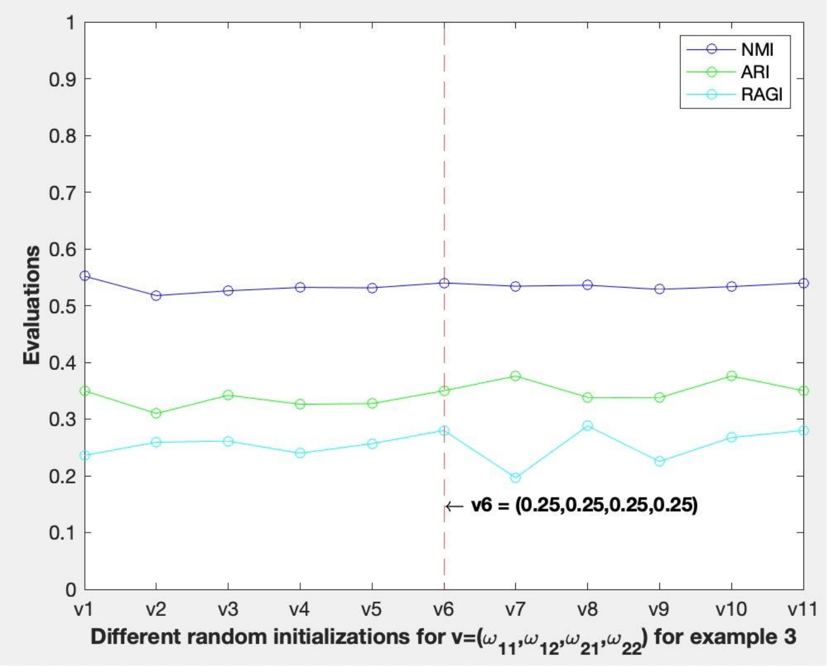
NMI
ARI
RAGI
Evaluations
← v6 = (0.25,0.25,0.25,0.25)
v1 v2 v3 v4 v5 v6 v7 v8 v9 v10 v11
Different random initializations for v=(ω_{11},ω_{12},ω_{21},ω_{22}) for example 3

(d)
