# Supplementary material for: scAWMV: an adaptively weighted multi-view learning framework for the integrative analysis of parallel scRNA-seq and scATAC-seq data
Source: Bioinformatics. 2022 Nov 16;39(1):btac739. doi: 10.1093/bioinformatics/btac739 (PMC9805575; doi:10.1093/bioinformatics/btac739)
Supplement: btac739_Supplementary_Data [file btac739_supplementary_data.zip › btac739_Supplementary_Data/supp.zip!/supp/tp3.pdf]

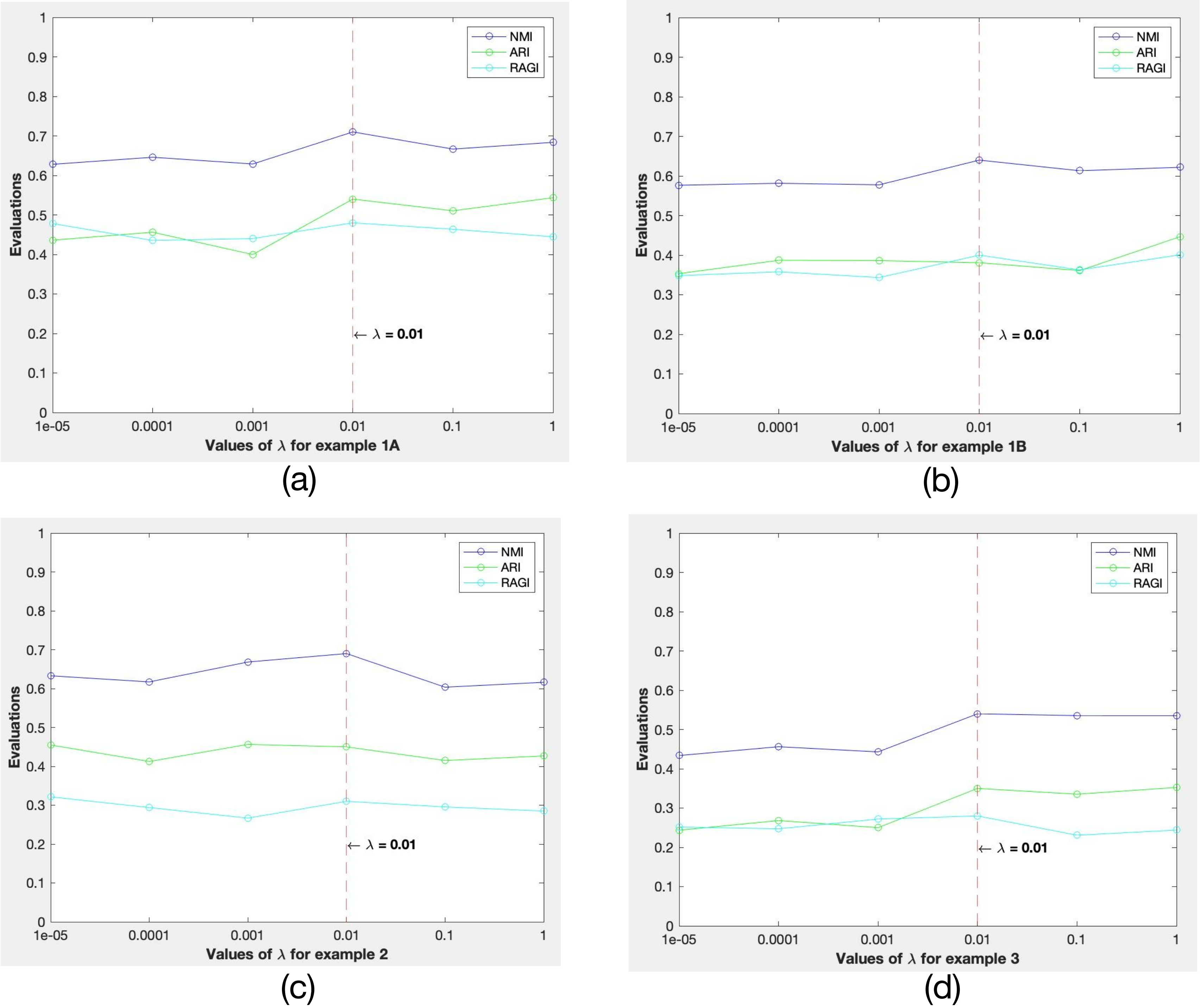

NMI
ARI
RAGI
Evaluations
← λ = 0.01
Values of λ for example 1A
(a)
Values of λ for example 1B
(b)
Values of λ for example 2
(c)
Values of λ for example 3
(d)
