# Supplementary material for: scAWMV: an adaptively weighted multi-view learning framework for the integrative analysis of parallel scRNA-seq and scATAC-seq data
Source: Bioinformatics. 2022 Nov 16;39(1):btac739. doi: 10.1093/bioinformatics/btac739 (PMC9805575; doi:10.1093/bioinformatics/btac739)
Supplement: btac739_Supplementary_Data [file btac739_supplementary_data.zip › btac739_Supplementary_Data/supp.zip!/supp/tp4.pdf]

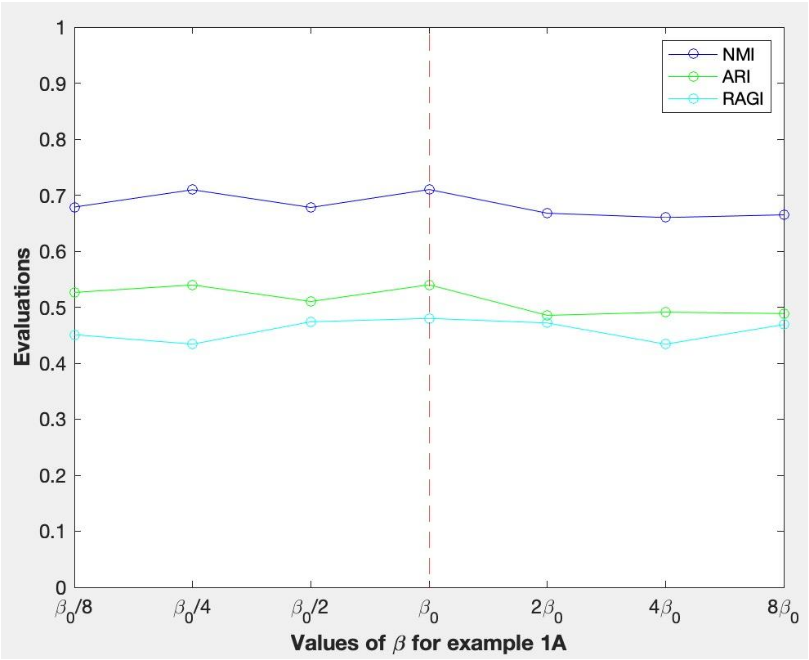
NMI
ARI
RAGI
Evaluations
0
0.1
0.2
0.3
0.4
0.5
0.6
0.7
0.8
0.9
1
$\beta_0/8$
$\beta_0/4$
$\beta_0/2$
β_0
$2\beta_0$
$4\beta_0$
$8\beta_0$
Values of β for example 1A
(a)

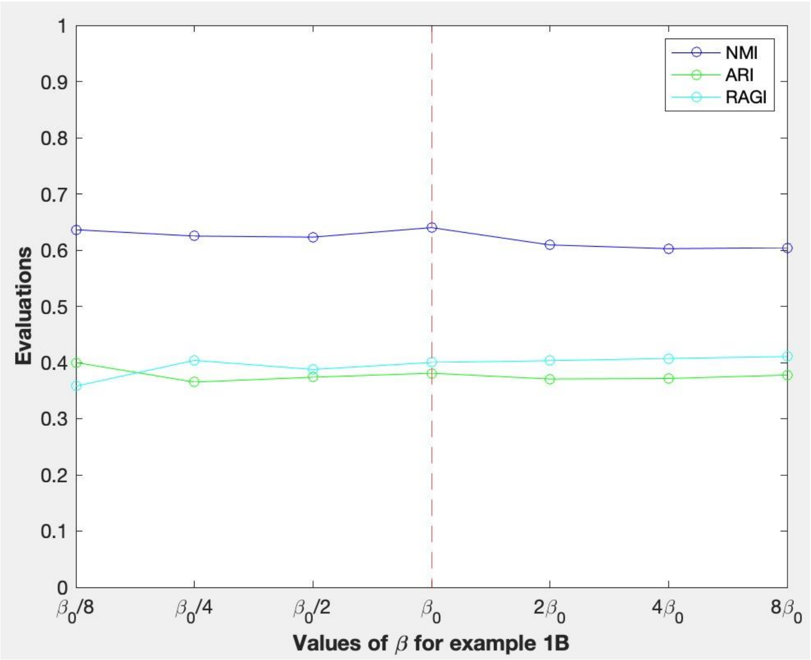
NMI
ARI
RAGI
Evaluations
0
0.1
0.2
0.3
0.4
0.5
0.6
0.7
0.8
0.9
1
$\beta_0/8$
$\beta_0/4$
$\beta_0/2$
β_0
$2\beta_0$
$4\beta_0$
$8\beta_0$
Values of β for example 1B
(b)

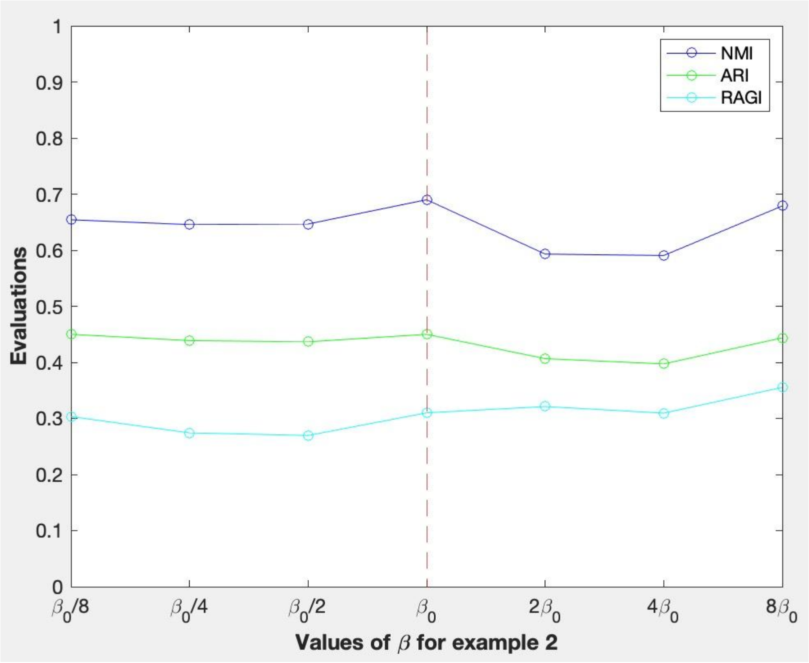
NMI
ARI
RAGI
Evaluations
0
0.1
0.2
0.3
0.4
0.5
0.6
0.7
0.8
0.9
1
$\beta_0/8$
$\beta_0/4$
$\beta_0/2$
β_0
$2\beta_0$
$4\beta_0$
$8\beta_0$
Values of β for example 2
(c)

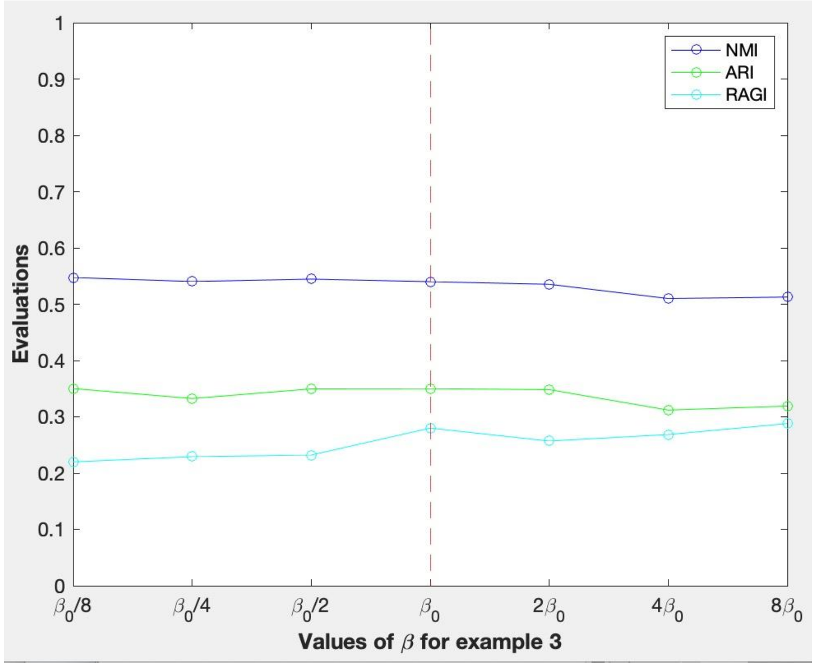
NMI
ARI
RAGI
Evaluations
0
0.1
0.2
0.3
0.4
0.5
0.6
0.7
0.8
0.9
1
$\beta_0/8$
$\beta_0/4$
$\beta_0/2$
β_0
$2\beta_0$
$4\beta_0$
$8\beta_0$
Values of β for example 3
(d)
