# Supplementary material for: scAWMV: an adaptively weighted multi-view learning framework for the integrative analysis of parallel scRNA-seq and scATAC-seq data
Source: Bioinformatics. 2022 Nov 16;39(1):btac739. doi: 10.1093/bioinformatics/btac739 (PMC9805575; doi:10.1093/bioinformatics/btac739)
Supplement: btac739_Supplementary_Data [file btac739_supplementary_data.zip › btac739_Supplementary_Data/supp.zip!/supp/tp5.pdf]

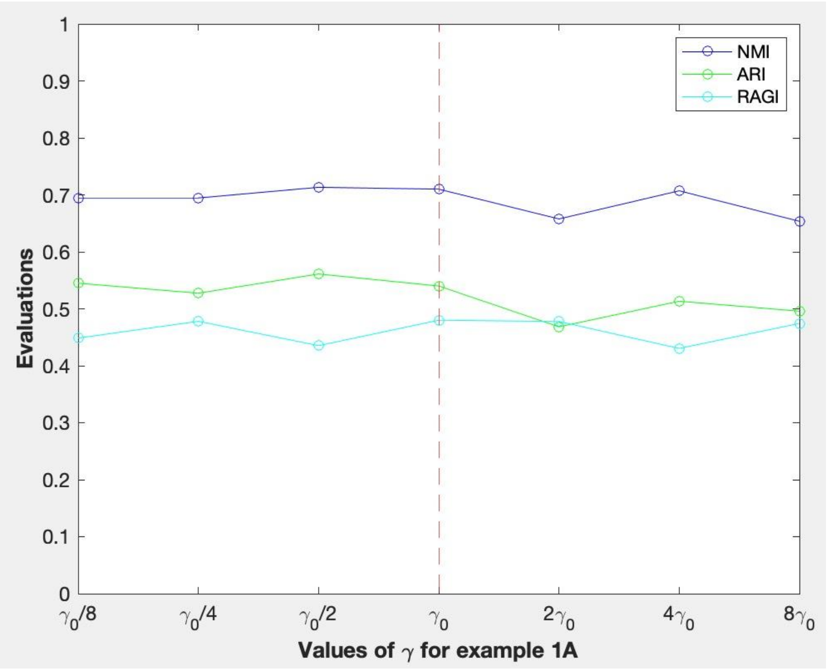
NMI
ARI
RAGI
Evaluations
0
0.1
0.2
0.3
0.4
0.5
0.6
0.7
0.8
0.9
1
$\gamma_0/8$ $\gamma_0/4$ $\gamma_0/2$ γ_0 $2\gamma_0$ $4\gamma_0$ $8\gamma_0$
Values of γ for example 1A
(a)

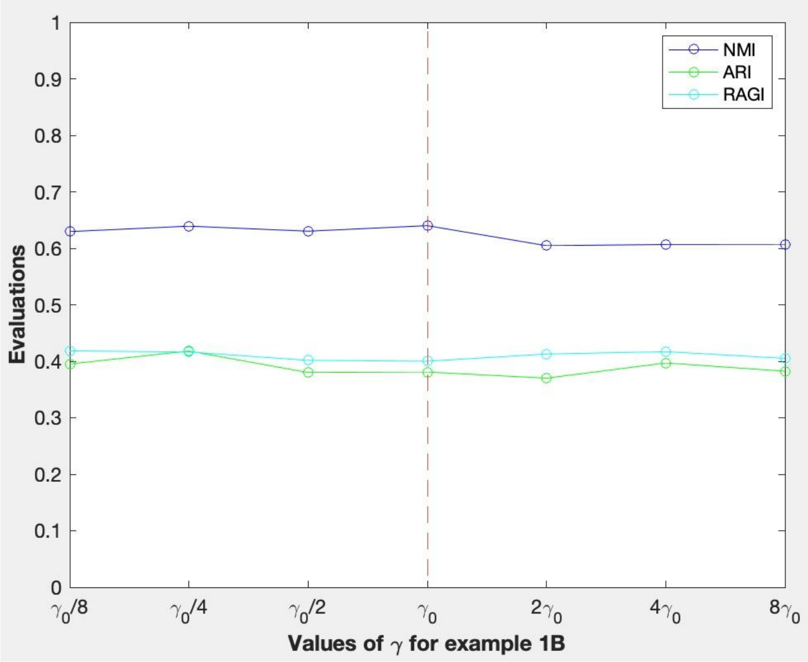
NMI
ARI
RAGI
Evaluations
0
0.1
0.2
0.3
0.4
0.5
0.6
0.7
0.8
0.9
1
$\gamma_0/8$ $\gamma_0/4$ $\gamma_0/2$ γ_0 $2\gamma_0$ $4\gamma_0$ $8\gamma_0$
Values of γ for example 1B
(b)

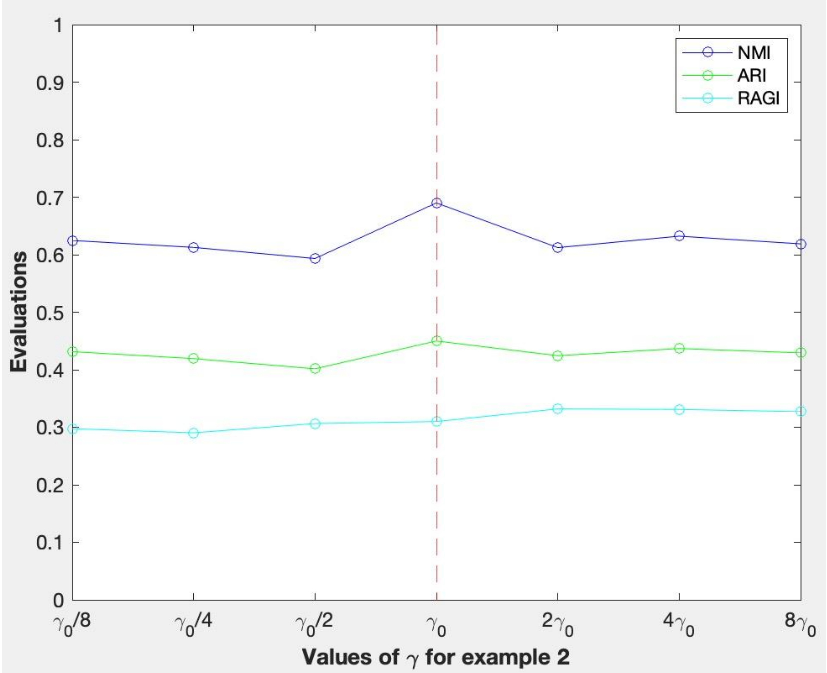
NMI
ARI
RAGI
Evaluations
0
0.1
0.2
0.3
0.4
0.5
0.6
0.7
0.8
0.9
1
$\gamma_0/8$ $\gamma_0/4$ $\gamma_0/2$ γ_0 $2\gamma_0$ $4\gamma_0$ $8\gamma_0$
Values of γ for example 2
(c)

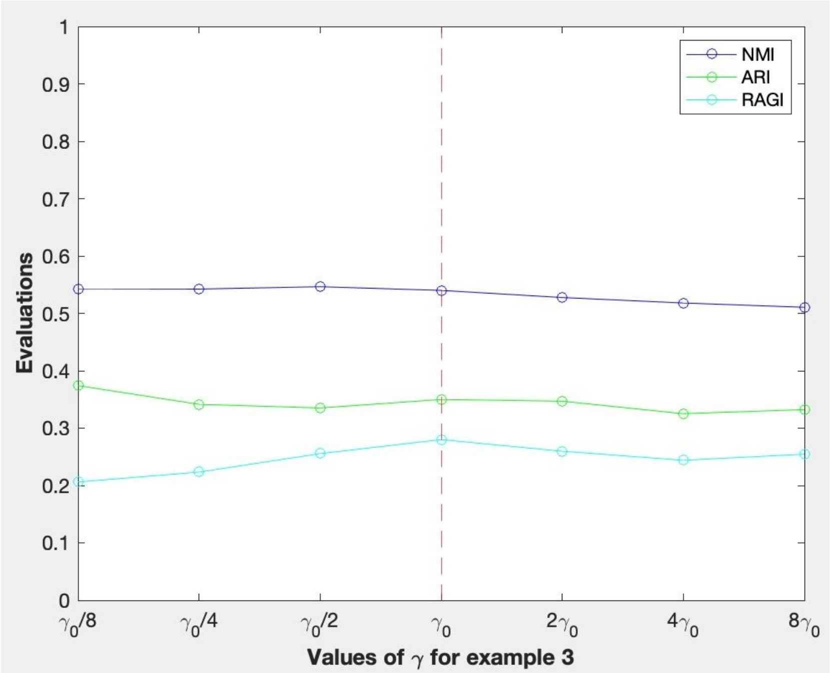
NMI
ARI
RAGI
Evaluations
0
0.1
0.2
0.3
0.4
0.5
0.6
0.7
0.8
0.9
1
$\gamma_0/8$ $\gamma_0/4$ $\gamma_0/2$ γ_0 $2\gamma_0$ $4\gamma_0$ $8\gamma_0$
Values of γ for example 3
(d)
