# Supplementary material for: scAWMV: an adaptively weighted multi-view learning framework for the integrative analysis of parallel scRNA-seq and scATAC-seq data
Source: Bioinformatics. 2022 Nov 16;39(1):btac739. doi: 10.1093/bioinformatics/btac739 (PMC9805575; doi:10.1093/bioinformatics/btac739)
Supplement: btac739_Supplementary_Data [file btac739_supplementary_data.zip › btac739_Supplementary_Data/supp.zip!/supp/tunepara1.pdf]

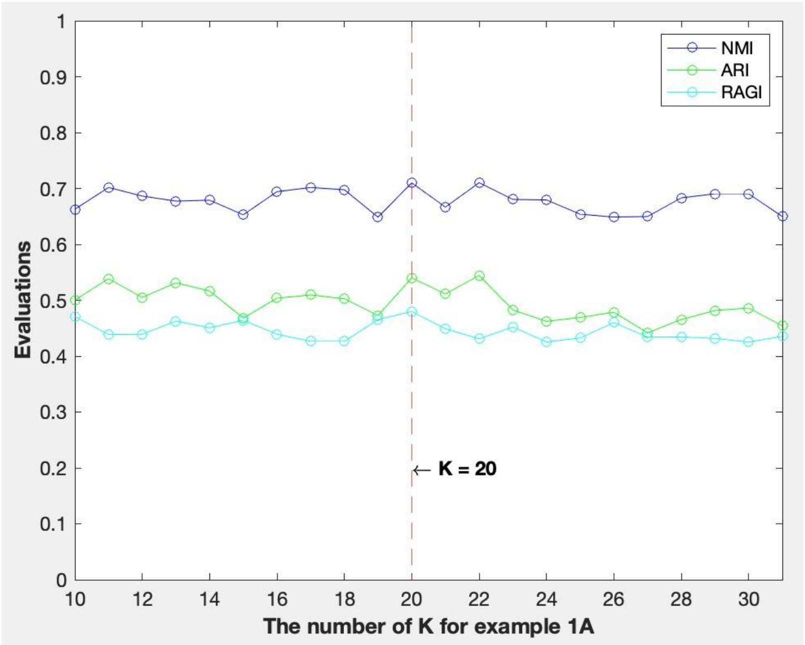

Evaluations
The number of K for example 1A
NMI
ARI
RAGI
← K = 20

(a)

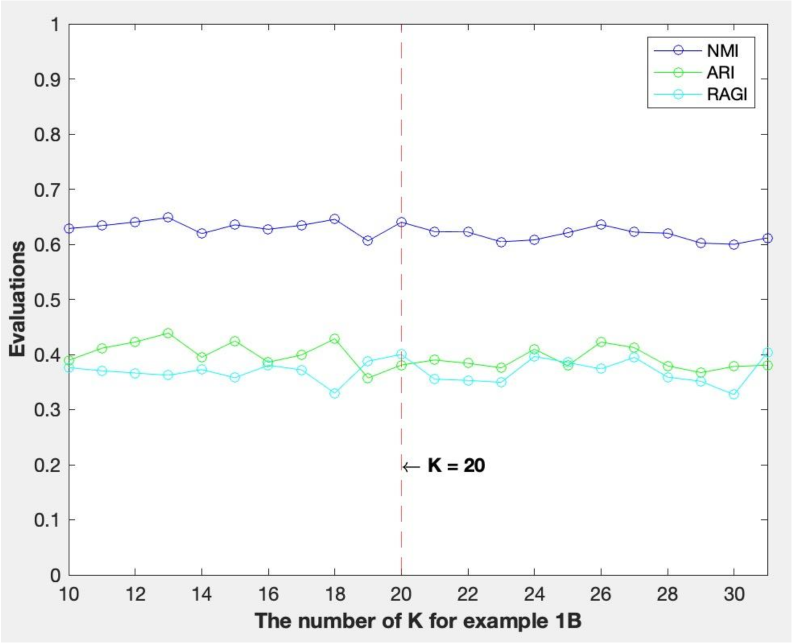

Evaluations
The number of K for example 1B
NMI
ARI
RAGI
← K = 20

(b)

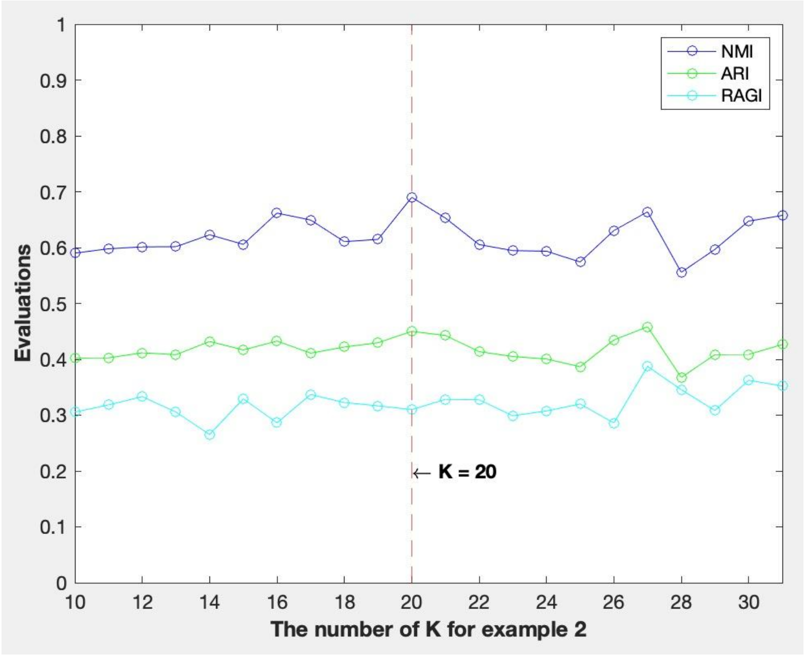

Evaluations
The number of K for example 2
NMI
ARI
RAGI
← K = 20

(c)

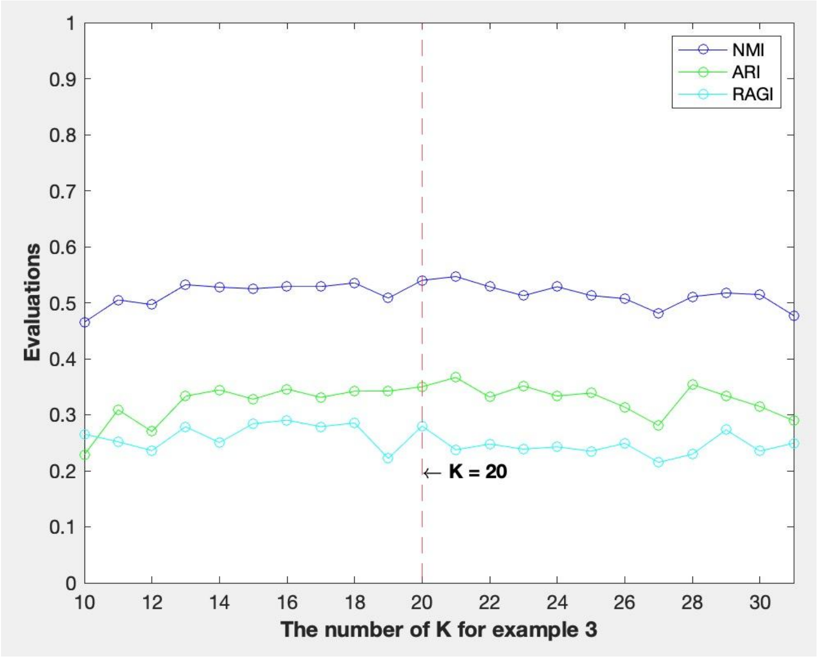

Evaluations
The number of K for example 3
NMI
ARI
RAGI
← K = 20

(d)
